# Topography-Driven Liquid Repellency in PFAS-Free Spherical-Particle Coating

**DOI:** 10.1021/acs.langmuir.6c03964

**Published:** 2026-10-14

**Authors:** Talya A. Scheff, Jinde Zhang, Hanna Dodiuk, Samuel Kenig, Margaret A. Auerbach, Timothy J. Lawton, Joey Mead

**Affiliations:** † 14710 University of Massachusetts, Lowell, Massachusetts 01854, United States; ‡ Shenkar College of Engineering and Art, Ramat Gan 5252626, Israel; § United States Army Combat Capabilities Development Command Soldier Center (DEVCOM SC), Natick, Massachusetts 01760, United States

## Abstract

The development of PFAS-free (per- and polyfluoroalkyl substances), liquid-repellent surfaces require a fundamental understanding of how surface geometry governs wetting-state stability. However, the independent role of interparticle spacing remains poorly understood because it is often coupled with changes in particle size, surface chemistry, or hierarchical morphology. Here, we present a geometry-driven strategy to systematically investigate the influence of spacing using re-entrant spherical architectures with controlled spacing ratios. Monodisperse 1 μm silica particles were immobilized within an epoxy matrix, while sacrificial 1 μm polystyrene spheres served as inert spacers to precisely tune interparticle distance without altering particle size or surface chemistry. After spacer removal and conformal hydrophobic silanization, wetting behavior was evaluated using ethanol–water mixtures with surface tensions ranging from 71.93 to 22.28 mN m^–1^. The apparent contact angle exhibits a nonmonotonic dependence on spacing ratio for liquids with surface tensions above approximately 25 mN m^–1^, reflecting a critical trade-off between reduced solid–liquid contact fraction, which enhances repellency, and diminished Cassie–Baxter state stability, which promotes wetting transition. As surface tension decreases, the optimal spacing ratio shifts toward smaller values, indicating increasingly stringent geometric requirements for sustaining the suspended wetting state. A theoretical framework integrating the Cassie–Baxter and Wenzel models with a breakthrough-pressure criterion quantitatively captures the experimental trends. These findings establish predictive design rules for geometry-controlled wetting and provide a scalable pathway for engineering high-performance, PFAS-free liquid-repellent coatings.

## Graphical abstract

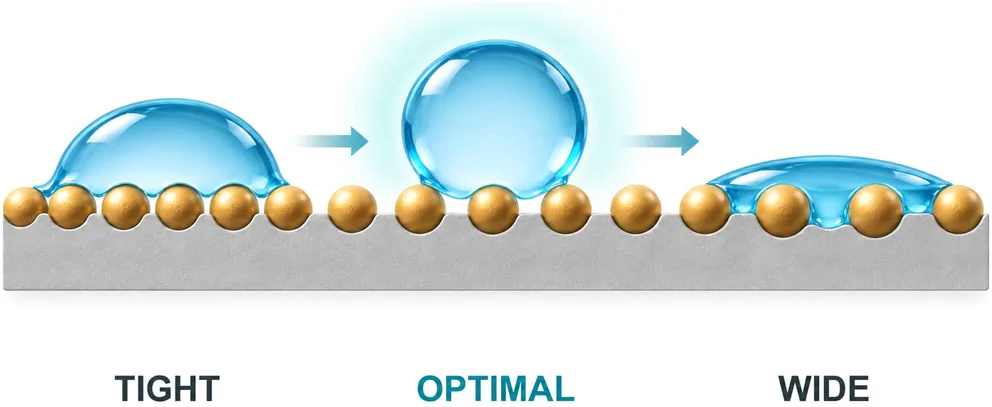

## Introduction

The ability to repel liquids across a broad range of surface tensions (omniphobicity) is central to applications in textiles,^1^ protective coatings,^2^ antifouling systems,^3^ and chemical barriers.^4^ For decades, per- and polyfluoroalkyl substances (PFAS) have dominated this field because of their exceptionally low surface energy and chemical inertness.^5–7^ The strong carbon–fluorine bond (≈485 kJ mol^–1^) reduces polarizability and intermolecular interactions, yielding surface energies as low as 6–20 mN m^–1^.^8^ As a result, PFAS-based surface treatments can repel not only water but also low-surface-tension liquids, enabling omniphobic performance in textiles and industrial materials.^5–7^

However, the same molecular stability that confers repellency also leads to extreme environmental persistence. PFAS compounds resist hydrolysis, photolysis, and biodegradation, leading to bioaccumulation and global contamination.^9^ Epidemiological studies have linked exposure to immune system effects, endocrine disruption, and carcinogenicity.^10^ Increasing regulatory restrictions and global phase-out initiatives have therefore intensified efforts to develop PFAS alternatives that maintain liquid repellency without fluorinated chemistry.^11^

Liquid repellency is fundamentally governed by wetting thermodynamics. The equilibrium contact angle of a liquid droplet on a smooth, homogeneous surface is described by Young’s equation^12^ (eq 1)­

1 $$\cos\theta_{Y}=\frac{\gamma_{SV}-\gamma_{SL}}{\gamma_{LV}}$$

where γ_SV_, γ_SL_, and γ_LV_ are the solid–vapor, solid–liquid, and liquid–vapor interfacial tensions, respectively. On a flat surface, reducing γ_SV_ via low-surface-energy chemistry increases θ_*Y*_.

However, Young’s equation cannot predict the contact angle on a surface that is rough and/or heterogeneous, such as a textile surface. For a rough surface with homogeneous chemistry, when liquid fully penetrates the asperities, its contact angle can be predicted by the Wenzel equation^13^ (eq 2), which extends Young’s equation by introducing the roughness factor *r*-

2 $$\cos\theta_{W}=r\cdot \cos\theta_{Y}$$

Where *r* represents the ratio of true to projected surface area, which is always greater than 1.

The Wenzel equation demonstrates that surface roughness amplifies intrinsic wettability: it enhances the hydrophobicity of naturally hydrophobic surfaces and the hydrophilicity of naturally hydrophilic ones. In Wenzel’s state, surface roughness cannot induce a wetting reversal (e.g., transforming a hydrophilic material into a hydrophobic one).^13^ Furthermore, the Wenzel state is characterized by the complete penetration of liquid into surface asperities. This results in significant contact angle hysteresis and high pinning forces, causing the liquid to adhere strongly to the surface- an outcome that is generally undesirable for achieving liquid repellency.^14,15^

Cassie and Baxter proposed an alternative wetting regime in which the droplet rests on a composite interface of solid and trapped air^16^ (eq 3)­

3 $$\cos\theta^{*}=\varphi_{S}\;\left(r_{\varphi }\;\cos\theta_{intrinsic}+1\right)-1$$

where φ_*S*_ is solid fraction defined as the area fraction of the liquid–air interface occluded by the texture and *r*_φ_ is the “roughness” of the wetted area, that is the actual wetted area compared to the occluded area. In contrast to the Wenzel state, the Cassie–Baxter state yields both a high apparent contact angle and low contact angle hysteresis, owing to the significantly reduced solid–liquid contact area. When the solid fraction (φ_*S*_) is sufficiently low (typically <0.5),^17^ the surface geometry can induce a wetting transition from intrinsic hydrophilicity to apparent hydrophobicity. This capability facilitates the development of omniphobic surfaces, which successfully repel liquids across a broad spectrum of surface tensions.

However, the Cassie–Baxter state is often metastable, especially for liquids with a low surface tension, and could have a nonreversible transition to the Wenzel state when downward pressure overcomes geometric resistance.^18,19^ Here, the downward pressure can be a combination of the droplet’s internal Laplace pressure, external hydrostatic pressure, or dynamic impact pressure.^19^ The “geometric resistance” refers to the antiwetting capillary pressure (often termed the critical breakthrough pressure, *P*_*c*_ *)* exerted by micro and nanostructures.^20^ These physical textures must pin the liquid–vapor interface to prevent the liquid from sagging or penetrating the air pockets.

For a simple cylindrical pore with characteristic spacing between structures, *D* (equivalent to the pore diameter), the antiwetting capillary pressure that the meniscus can withstand is classically described by the Young–Laplace eq 4 ^21^

4 $$P_{c}=\frac{-4\gamma_{LV}\;\cos\theta_{intrinsic}}{D}$$

where *D* is the spacing between structures (or pore diameter). θ_Intrinsic_ is the intrinsic contact angle of the liquid on a smooth, flat version of that solid. γ_LV_ is the liquid–vapor surface tension. If the liquid is water, its high surface tension combined with a hydrophobic coating usually results in θ_Intrinsic_ > 90^*°*^. Because the cosine of an angle greater than 90° is negative, the resulting *P*_*c*_ is positive, meaning the capillary force pushes upward, resisting intrusion. However, for oils or organic solvents, the intrinsic angle θ_Intrinsic_ is almost always <90°. The cosine becomes positive, making *P*_*c*_ negative. The capillary forces no longer resist the liquid; they actively suck the liquid down into the pores via spontaneous capillary action. If chemistry alone dictates that the liquid should wet the surface (θ_Intrinsic_ < 90°), the only way to generate an upward antiwetting capillary pressure is by radically altering the physical geometry of the surface textures. This is accomplished using re-entrant structures, topographical features where the physical geometry curves back inward beneath itself, creating an overhang or an undercut. In this case, the antiwetting capillary pressure will be described by eq 5 ^22^

5 $$P_{c}=\frac{-4\gamma_{LV}\;\cos{(\theta }_{intrinsic}+\theta_{geo})}{D}$$

Where θ_Geo_ is reentrant geometric angle, which is defined as the angle the overhanging wall makes relative to the vertical axis of the pillar.^23^ Several re-entrant structures are illustrated in Figure S1.

Inclusion of θ_Geo_ inside the cosine function is the mathematical key to omniphobicity. It dictates how the physical structure manipulates the local contact angle of the meniscus at the pinning edge. For a standard straight pillar (θ_Geo_ = 0°), the wall is vertical. eq 5 reduces to eq 4. If you introduce a liquid with θ_Intrinsic_ = 60°, *cos* (60°) is positive, making the entire *P*_*c*_ negative. The capillary pressure pulls the liquid down, causing Wenzel wetting. However, for T-Shaped re-entrant pillar (θ_Geo_ = 90°), the pillar has a horizontal overhang. If you apply the exact same liquid, the angle inside the cosine becomes cos­(60° + 90°) = cos (150°), which is −0.866 and it cancels the negative sign at the front of eq 5. *P*_*c*_ becomes heavily positive, generating a strong upward restoring force that suspends the low-surface-tension liquid in the Cassie state.

Biological systems provide clear paradigms for such topography-driven liquid repellency. The lotus leaf relies on hierarchical micro- and nanoscale roughness to stabilize trapped air plastrons, yielding superhydrophobicity, super-repellent to water.^24^ However, due to a lack of re-entrant topographical features, the lotus leaf architecture is highly susceptible to wetting by low-surface-tension liquids.^25^ Conversely, the springtail cuticle utilizes complex, re-entrant nanocavities that successfully repel both aqueous and organic liquids, maintaining a Cassie–Baxter state even under elevated hydrostatic pressure.^26^ These contrasting examples demonstrate that while chemical composition is foundational, geometric design is the decisive factor in realizing true omniphobicity.

Engineering omniphobic surfaces has therefore focused on combining low-surface-energy chemistry with re-entrant architecture. Tuteja et al. demonstrated robust omniphobicity by integrating fluorodecyl-POSS chemistry with re-entrant macrotextures, achieving repellency down to γ ≈ 27.3 *mN m* ^*–*1^.^27^ Bhushan et al. derived empirical stability criteria showing that the Cassie state can be maintained when geometric spacing satisfies approximately 4*R* < *D* < 20*R* where *R* is the radius of the spherical elements.^28^ Liu and Kim introduced doubly re-entrant microposts that repel even fully wetting liquids through purely geometric design principles, demonstrating that geometry can compensate for limited surface chemistry.^29^ Panter et al. performed computational energy barrier optimization and identified optimal solid fractions φ_*s*_ ≈ 0.04–0.09, corresponding to spacing ratios *D*/*R* ≈ 5–15.^30^ More recently, Wong et al. reported fluorine-free hierarchical coatings capable of repelling liquids with a contact angle of ∼30 mN m^–1^, highlighting the potential of morphology-driven design without PFAS.^31^ Recent efforts toward developing PFAS-free omniphobic surfaces have focused on combining surface texture with alternative low-surface-energy chemistries to achieve liquid repellency without fluorinated compounds. A variety of strategies have been explored, including silicone-based coatings^32^ and hydrocarbon-based coatings,^33^ as well as lubricant-infused surfaces^34^ and hierarchically structured surfaces,^35^ which enable the repellency of a broad range of liquids by stabilizing a composite liquid–air interface. These approaches rely on carefully engineered surface topographies to sustain the Cassie–Baxter state^36^ and suppress liquid penetration through geometric effects^37^ and capillary pressure considerations,^38^ thereby enabling omniphobic behavior in the absence of fluorinated chemistries. Among these strategies, particle-based systems—particularly those utilizing spherical silica particles - have emerged as a promising platform, as they provide tunable roughness and enable the formation of re-entrant geometries that enhance liquid repellency through purely geometric effects.^39^

Nano- and microscale spherical particles offer a highly attractive platform for designing re-entrant, topography-driven omniphobic surfaces, effectively bridging geometric idealities with practical scalability.^39^ Unlike complex re-entrant microstructures that require specialized, costly fabrication, monodisperse spherical particles are commercially abundant and compatible with scalable, low-cost coating/finishing techniques.^33^ Spray coating offers a simpler, scalable deposition route; however, the present sacrificial-spacer method is used as a controlled model system in which interparticle spacing can be varied while particle size and surface chemistry remain constant. From a geometric perspective, spheres inherently provide a severe re-entrant structural angle that can approach 90°. Based on established thermodynamic stability criteria, such large re-entrant angles are essential for suspending liquids with low intrinsic contact angles and thus low surface tensions in a robust Cassie–Baxter state. While these characteristics strongly suggest that spherical assemblies can achieve broad repellency without sacrificing manufacturability, the fundamental physical limits of such architectures remain systematically unexplored. Current literature predominantly focuses on isolated, optimized geometries rather than systematically mapping structural parameters to liquid surface-tension thresholds.^30^ Notably lacking is a comprehensive design framework that correlates interparticle spacing with the critical surface tension required to trigger the Cassie-to-Wenzel transition within fluorine free spherical architectures. Furthermore, prior investigations typically rely on a restricted subset of test liquids, leaving the broad spectrum of surface tensions largely unexplored.

To address this gap, we developed a systematic, topography-controlled platform using 1-μm silica spheres in an epoxy binder, employing a sacrificial polystyrene spacer method to tune the interparticle distance (2*D*) and normalized spacing ratio (D*=*(D+R)*/*R* where D* represents the center-to-center particle spacing normalized by particle diameter) across the range of 1 < D* < 35.^27^ Wetting performance was evaluated across a wide range of surface tensions (71.93 to 22.28 mN m^–1^) using ethanol–water mixtures, thoroughly probing Cassie stability across the omniphobic regime. By correlating the critical spacing ratio with the liquid surface tension, we established a predictive framework for topography-driven liquid repellency independent of fluorinated chemistry.^30^ Rather than treating liquid repellency as a purely chemical phenomenon, we reframe it as a fundamental competition between downward liquid pressure and geometric resistance.^20^ This perspective enables the rational design of PFAS-free surfaces precisely tailored to specific liquid repellency requirements.

## Experimental Section

### Materials

Epoxy resin (Epoxy technology, EPO-TEK 301, two-part epoxy system parts A and B) was used as the binder matrix. Monodisperse silica spheres with a nominal diameter of 1 μm (Bangs Laboratories, Inc., SS04000, 10 wt % aqueous suspension) were used as the primary structural elements. Monodisperse polystyrene (PS) spheres with a nominal diameter of 1 μm (Bangs Laboratories, Inc., PS04001, 10 wt % aqueous suspension) were used as sacrificial inert spacer particles to control interparticle spacing. Toluene (purity ≥ 99.5%, Sigma-Aldrich) and hexane (purity ≥ 95–99%, Sigma-Aldrich) were used as solvents for the removal of the PS spheres. Hexadecyltrimethoxysilane (HDTMS, purity ≥ 85%, Sigma-Aldrich) was used to functionalize hydrophobic surfaces. Isopropanol (IPA, purity ≥ 99.5%, Sigma-Aldrich), ethanol (purity ≥ 99.5%, Sigma-Aldrich), acetic acid (purity ≥ 99.7%, Sigma-Aldrich), and deionized water (18.2 MΩ·cm) were used as received without further purification.

The silica and PS particles were supplied as commercially available aqueous suspensions and were used as received; no additional surfactant or dispersing agent was added.

### Coating Preparation

The fabrication of silica particle arrays with systematically controlled interparticle spacing is illustrated in Figure 1. Glass substrates were initially cleaned with isopropanol (IPA) and coated with epoxy resin to form a uniform adhesive layer. To optimize particle anchoring and partial embedding, the epoxy was precured at 110 °C for 4–10 min, rendering the surface appropriately tacky. Subsequently, the substrates were dip-coated in an aqueous binary suspension containing monodisperse 1-μm silica spheres (structural elements) and 1-μm polystyrene (PS) spheres (sacrificial inert spacers). After particle deposition, the samples were dried and subjected to a final thermal cure at 110 °C for 2 h, resulting in the immobilization of most embedded spheres within the cross-linked epoxy matrix. The PS spacers were then removed via selective dissolution in toluene, hexane, or a toluene–hexane mixture. This process selectively etched the PS spacers without compromising the silica spheres or the epoxy binder. Finally, the resulting topography was functionalized with hexadecyltrimethoxysilane (HDTMS) introducing a hydrophobic surface chemistry.

**Figure 1:**
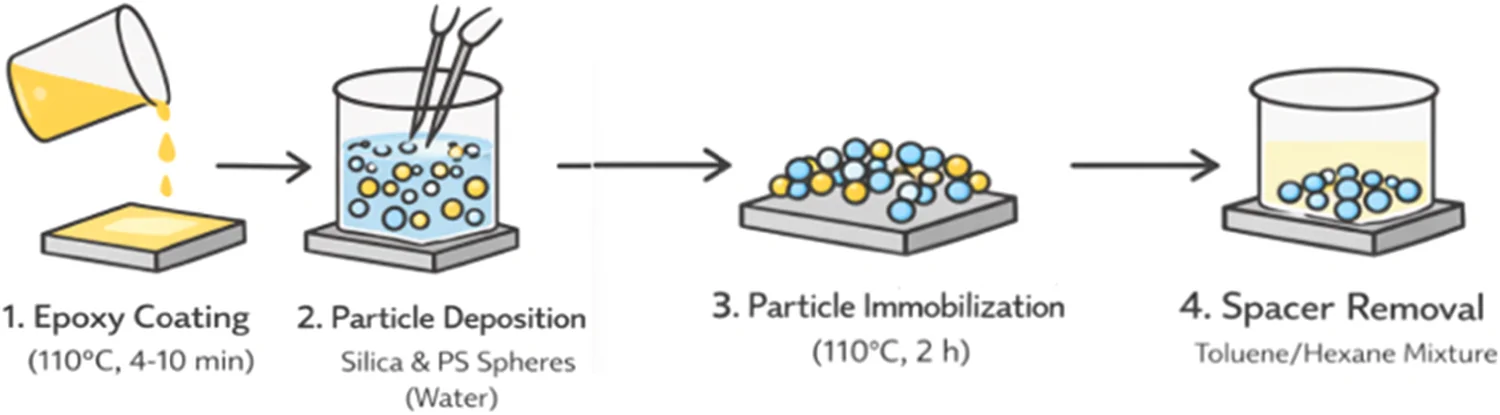
Fabrication Process for Spherical Particle Coatings with Controlled Interparticle Spacing Using a Sacrificial Spacer Approach. Blue spheres represent silica structural particles, and yellow spheres represent sacrificial PS spacer particles.

Before dip coating, the selected volumes of the 10 wt % PS and silica suspensions were combined at the required PS/silica suspension-volume ratio and sonicated for 5 min immediately before use. After spacer removal, the structured samples and the smooth glass reference were dipped in a silane-treatment solution containing 1.0 g HDTMS, 50.0 g IPA, 2.0 g water, and 1.8 g acetic acid (pH approximately 4), followed by heating at 120 °C for 30 min.

### Characterization

Surface topography was characterized using scanning electron microscopy (SEM, JEOL JSM-7401F field-emission SEM, accelerating voltage 4–10 kV) before and after removal of the polystyrene (PS) spacer particles to evaluate particle distribution, embedding depth, and the resulting interparticle spacing. Energy-dispersive X-ray spectroscopy (EDX; Oxford Instruments X-Max detector attached to the SEM) was used to distinguish silica spheres (Si signal) from PS spheres (C signal) and to confirm the removal of PS after solvent treatment. The solvent used for spacer removal was also collected, deposited on a glass slide, and analyzed by EDX to verify the composition of the removed material. Wettability was evaluated using static contact angle measurements with a goniometer (KRÜSS DSA100 Drop Shape Analyzer) using ethanol–water mixtures with surface tensions ranging from 71.93 to 22.28 mN m^–1^ and the volume of the droplet was 6 μL. For each condition, 5–6 measurements were performed at different locations on the sample surface, and the resulting average values were used to generate the error bars.

The intrinsic contact angle used as the smooth-surface reference was measured on HDTMS-treated glass prepared using the same silane treatment as the particle-coated samples. Water/ethanol compositions and target surface tensions were taken from literature values; the mixtures were prepared by weight and checked by pendant-drop analysis at room temperature before use. (Table S1). Additionally, representative sessile-drop images and additional advancing/receding contact-angle measurements are provided in the Supporting Information (Figures S10–S13)

## Results and Discussion

### Formation of Controlled Interparticle Spacing via a Sacrificial Spacer

To optimize particle anchoring and partial embedding, the epoxy was precured at 110 °C for varying durations to tune the coating’s tackiness and viscosity. This precuring step governs the balance between particle adhesion and exposure, which is critical for achieving the desired surface roughness and re-entrant geometry. Details of this optimization study, including the effect of precuring time on particle embedding depth and surface morphology, are provided in the Supporting Information (Figures S3–S5). The optimized precuring time, 6 min, was used in all the experiments reported in this paper.

To control the spacing between silica particles, 1-μm polystyrene (PS) spheres were used as sacrificial spacers and codeposited with 1-μm silica spheres onto the precured epoxy surface. Following curing, PS was selectively dissolved in toluene, hexane, or their mixtures. As a result, the volume previously occupied by PS spheres became the interparticle space between silica spheres. By tuning the ratio of PS spheres to silica spheres in the formulation, different interparticle spacing was achieved. The topography before and after solvent treatment is shown in Figure 2. Prior to PS removal, the surface contains a mixed population of spheres as seen in Figure 2a. Because both the PS and silica spheres used in this study have a diameter of 1 μm, distinguishing between them in standard scanning electron microscopy (SEM) images is challenging. However, following the selective removal of the PS spheres, the surface topography changes distinctly: discrete voids appear at the positions (Figure 2b).

**Figure 2:**
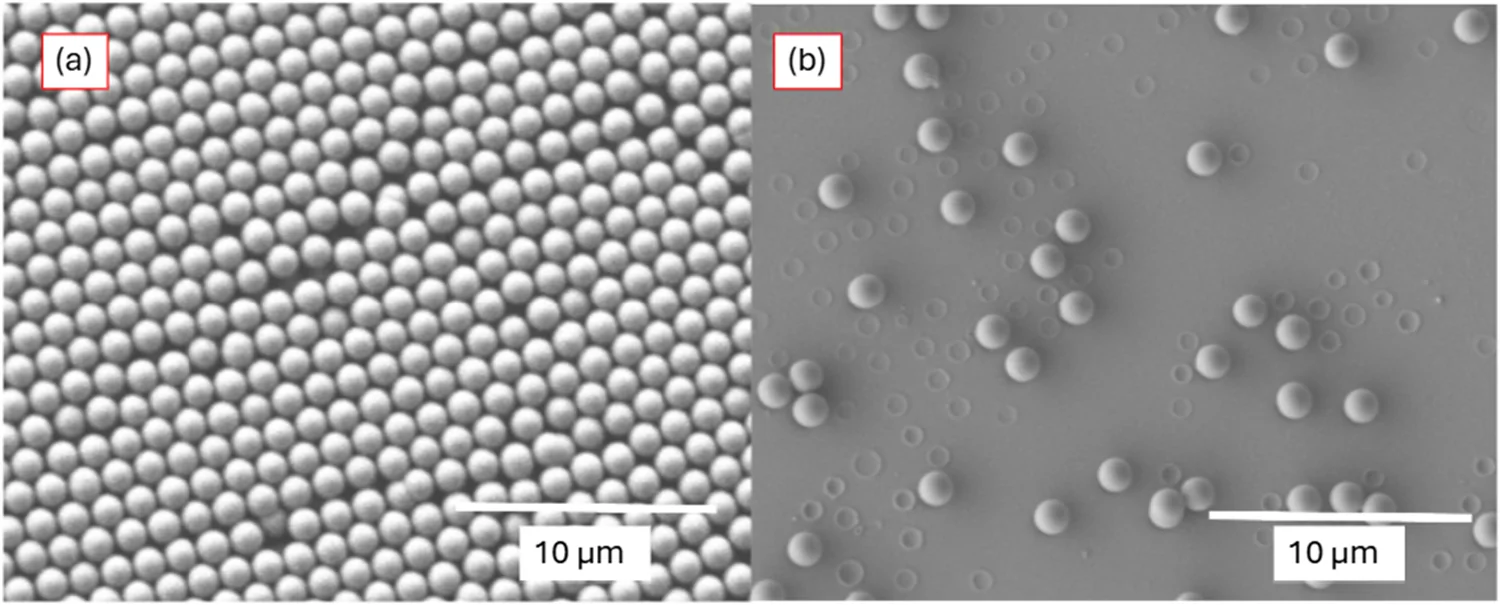
SEM images of the representative coating (a) before and (b) after sacrificial PS spacer removal, showing the formation of interparticle spacing. The sample was prepared with a 3:1 PS/silica suspension-volume ratio, and the PS spacer was removed using a 1:1 hexane:toluene mixture for 2 min.

To further validate the efficacy of the sacrificial spacer method, energy-dispersive X-ray spectroscopy (EDX) mapping was performed in tandem with SEM before and after spacer removal (Figure 3). Prior to solvent treatment (Figure 3a), the carbon signal corresponds to both the PS spheres and the epoxy matrix, while the silicon signal identifies the silica spheres. The coexistence of distinct carbon-rich and silicon-rich spherical features confirms the presence of both particle types. After solvent treatment (Figure 3b), the EDX maps reveal that the remaining spherical features are exclusively silicon-rich, confirming they are silica particles. (Detailed EDX results regarding the signal of carbon and silica are provided in the Supporting Information Figure S8). The carbon signal is now uniformly associated with the underlying epoxy surface rather than discrete spherical structures. This elemental analysis confirms the complete removal of the PS spheres, while some of silica particles might also be washed off with PS, which was indicated by the much smaller amount of silica particles in Figure 3b compared to Figure 3a. To determine whether the solvent treatment used to remove the sacrificial polystyrene phase also caused the detachment of silica spheres, the solvent collected after immersion was analyzed via SEM/EDX. The wash solution was deposited onto a clean glass substrate, allowed to dry, and the remaining material was examined by EDX, as seen in Figure S6. The elemental maps show discrete spherical features that appear in yellow, corresponding to the silicon signal. These yellow spherical regions confirm the presence of silica particles that were washed off during the solvent treatment.

**Figure 3:**
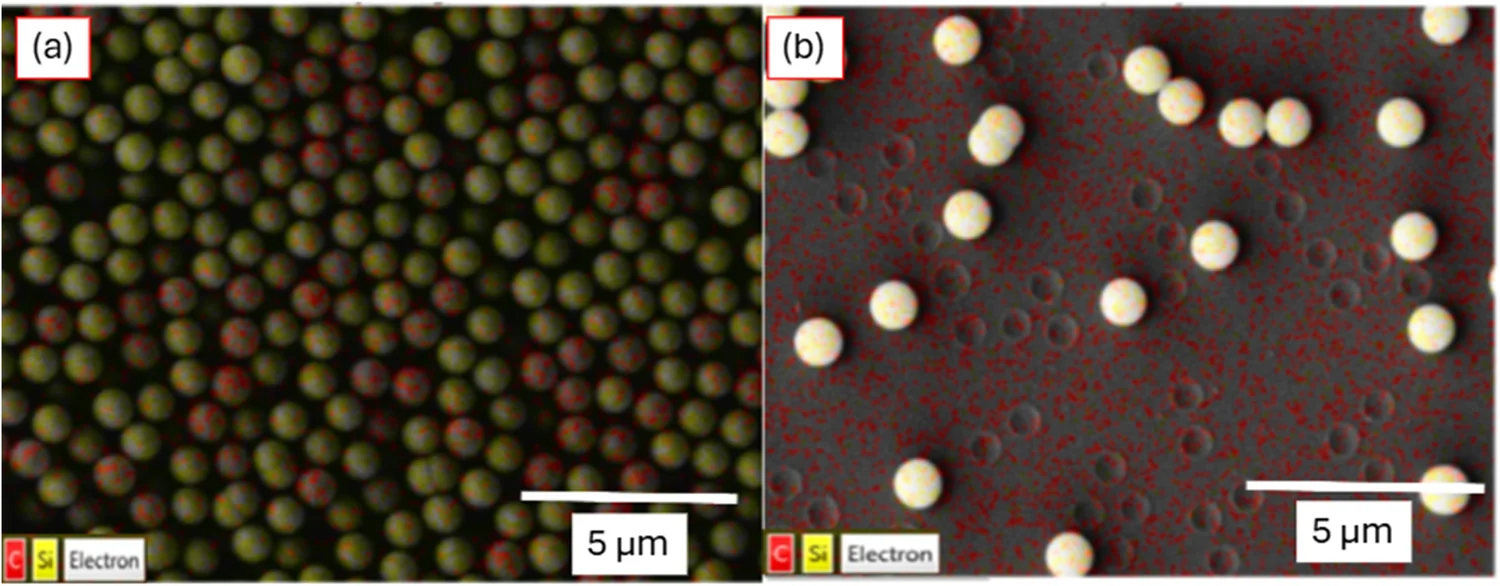
EDX Elemental Mapping Confirming Selective Removal of Polystyrene Spacer Particles (a) before and (b) after PS removal.

### Generation of Controlled Spacing Configurations

Following the validation of the inert spacer removal and the structural composition of the remaining scaffold, our next objective was to generate a range of interparticle distances. This was achieved by systematically varying key processing parameters, specifically the PS-to-silica ratio and the solvent composition used during selective dissolution. By adjusting these variables, multiple surface configurations were fabricated with varying interparticle spacing, while maintaining a constant particle diameter (1 μm) and identical surface chemistry. The silica spheres remained monodisperse and largely retained within the epoxy matrix across all conditions, ensuring that spacing was the primary structural variable. Detailed work regarding effects of the PS-to-silica ratio on the resulting spacing is provided in the Supporting Information (Figure S7). Representative SEM micrographs of these architectures are presented in Figure 4, clearly illustrating progressively increasing interparticle spacing across the different samples. Quantification of the interparticle spacing was performed using measurements collected from several SEM images acquired at different representative regions across each surface to ensure that the analyzed morphologies accurately reflected the overall surface structure and particle distribution (see Supporting Information, Figure S7). To determine the spacing ratio, D*, SEM images were processed into binary formats using ImageJ software. The contrast thresholds were manually adjusted to accurately segment the morphological features, with black pixels representing the microspheres and white pixels representing the interstitial void space. The software was then utilized to calculate the area ratio of the black and white regions, which was subsequently converted to derive the average spacing ratio, D*. The resulting spacing configurations were designated as D1–D6, corresponding to progressively increasing spacing ratios calculated using ImageJ image analysis software.

**Figure 4:**
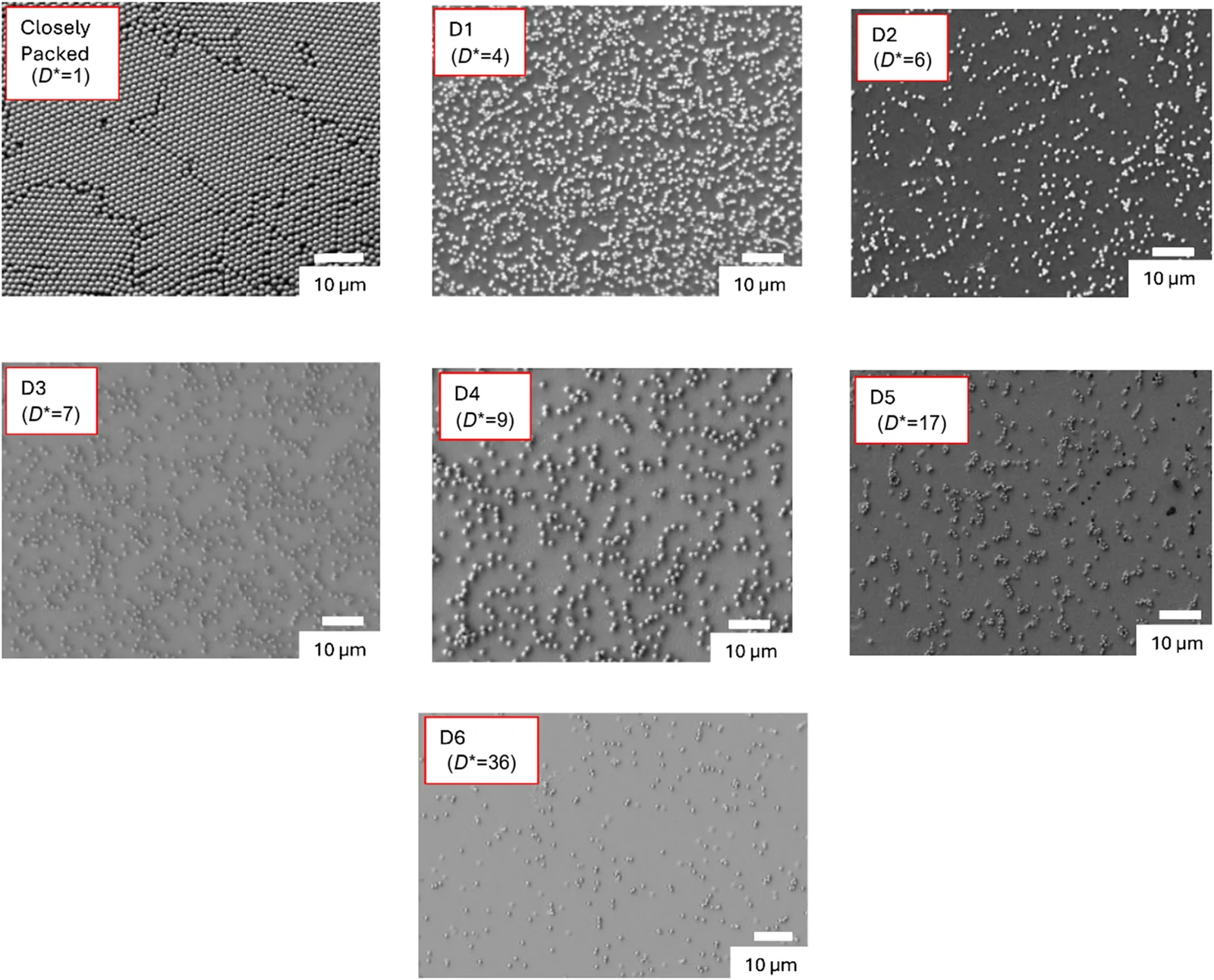
SEM Images of Surfaces with Varied Interparticle Spacing Ratios.

The SEM images show dispersed individual particles without obvious large-scale agglomeration. Because the particle arrangement is stochastic, the reported D* values represent average normalized spacing ratios obtained from representative SEM regions rather than a perfectly periodic spacing.

### Spacing-Dependent Repellency across a Continuous Surface Tension Range

To evaluate the liquid repellency of silica spheres with varying spacing ratios (*D* ^*^), contact angle measurements were performed using ethanol–water mixtures with surface tensions ranging from 71.93 mN m^–1^ (water) to approximately 22.37 mN m^–1^ (ethanol). The performance map summarizing these results is presented in Figure 5.

**Figure 5:**
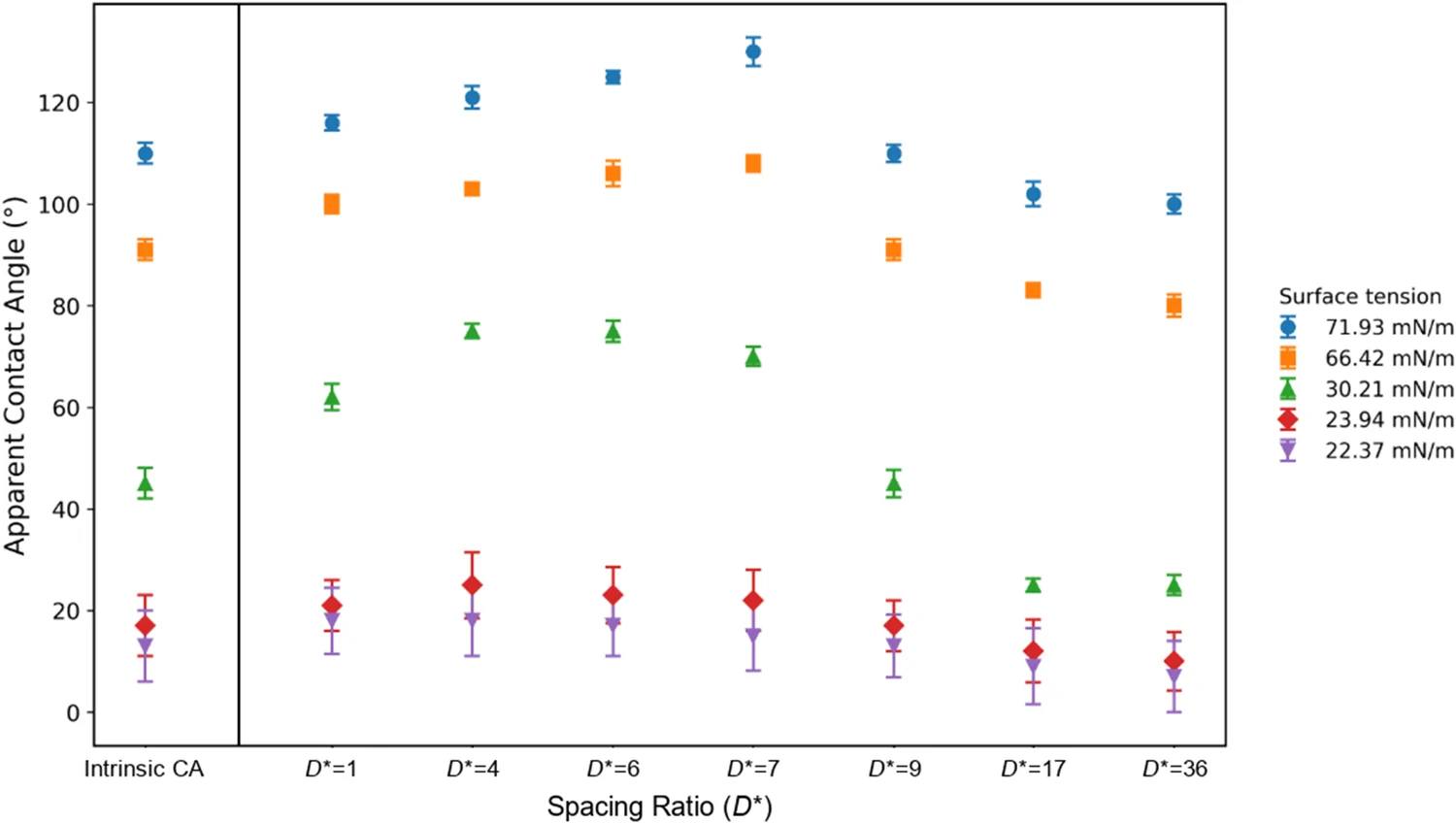
Apparent Contact Angle as a Function of Spacing Ratio for Polar Ethanol–Water Mixtures with Varying Surface Tension. All structured samples were HDTMS-treated after PS removal; the intrinsic values at the left correspond to smooth HDTMS-treated glass.

The wetting behavior of liquids on the fabricated spherical arrays is strongly dependent on both the intrinsic liquid surface tension and the normalized interparticle spacing ratio (D*). For liquids with moderate to high surface tensions (30–70 mN m^–1^) (θ_Intrinsic_, 40° – 110°), the apparent contact angle demonstrates a distinct nonmonotonic trend. Initially, as D* expands from near-close-packed configurations, the apparent contact angle rises significantly above the intrinsic baseline established on a smooth, identically functionalized surface. This enhancement is consistent with a metastable Cassie–Baxter-like state; increasing the interparticle gap effectively minimizes the solid area fraction at the liquid–solid interface, thereby favoring a reduced solid–liquid contact fraction and droplet suspension even for liquids with intrinsic angles below 90°. However, this topography-driven repellency reaches a distinct geometric and thermodynamic limit. For these higher-surface-tension liquids, apparent contact angles peak at an optimal spacing ratio of approximately D* = 6–7. Beyond this, further widening of the interparticle gaps critically compromises the stability of the Cassie–Baxter state. The interfacial meniscus can no longer span the separated spheres, triggering localized sagging and spontaneous capillary intrusion. This initiates a progressive Cassie-to-Wenzel transition, evidenced by a sharp decline in the apparent contact angle. Most notably, at large spacing ratios (D* > 9), the apparent contact angles systematically drop below their corresponding intrinsic values, which is consistent with increased liquid penetration and Wenzel-like wetting.

Static contact angle alone does not directly prove a Cassie–Baxter state. However, for liquids with intrinsic contact angles below 90°, the Wenzel relation predicts that roughness should further decrease the apparent contact angle, whereas the measured apparent angle increases above the intrinsic value at intermediate spacing. We therefore interpret this region as consistent with a Cassie–Baxter-like composite interface, supported by the re-entrant spherical geometry and the calculated robustness trend.

Conversely, the experimental data in Figure 5 reveal strict performance boundaries for liquids with exceptionally low surface tensions (exhibiting θ_Intrinsic_ < 20°). Unlike liquids with higher surface tension, these liquids display a negligible response to structural modulation, maintaining a highly wetted Wenzel state irrespective of the spacing ratio. For such high wetting fluids, the antiwetting capillary pressure generated by the spherical geometry is fundamentally insufficient to overcome the thermodynamic driving force for intrusion. In our case, the driving force mainly came from the Laplace internal pressure inside the liquid droplet.

In addition to the nonmonotonic behavior illustrated in Figure 5, we observe a systematic shift in the critical spacing ratio (D*) at which the apparent contact angle is maximized. Specifically, as the liquid surface tension decreases, this critical spacing shifts toward smaller values (i.e., the peak of the curve moves to the left). This indicates that lower-surface-tension liquids require tighter interparticle spacing to maintain a robust Cassie–Baxter state. This phenomenon can be explained by eqs 1 and 4. According to Young’s eq (eq 1), lower-surface-tension liquids inherently exhibit smaller intrinsic contact angles (θ_Intrinsic_), a trend confirmed by our baseline measurements on flat silane-treated glass (Figure 5). Furthermore, eq 4 establishes that the critical breakthrough pressure (*P*_*c*_) which dictates the robustness of the Cassie–Baxter state decreases alongside surface tension (γ_LV_) and intrinsic contact angle (θ_Intrinsic_), but scales inversely with the interparticle spacing (*D*). Therefore, to maintain a sufficiently high *P*_*c*_ to suspend a lower-surface-tension liquid, the structural gap must be reduced. This theoretical requirement perfectly aligns with our experimental observations. It should be noted that the liquids investigated in this study were exclusively polar ethanol–water mixtures, and therefore the observed spacing-dependent wetting behavior has not yet been verified for nonpolar liquids such as oils.

To visualize this trend more clearly, Figure 6 plots the spacing ratio at which the maximum apparent contact angle occurs as a function of surface tension. This representation isolates the transition point for each liquid and removes the complexity of the previously shown full performance curves. Figure 6 clearly demonstrates that as surface tension decreases, the spacing ratio corresponding to peak performance shifts toward smaller values. In other words, more wetting liquids require a closer-packed morphology to achieve their maximum attainable apparent contact angle.

**Figure 6:**
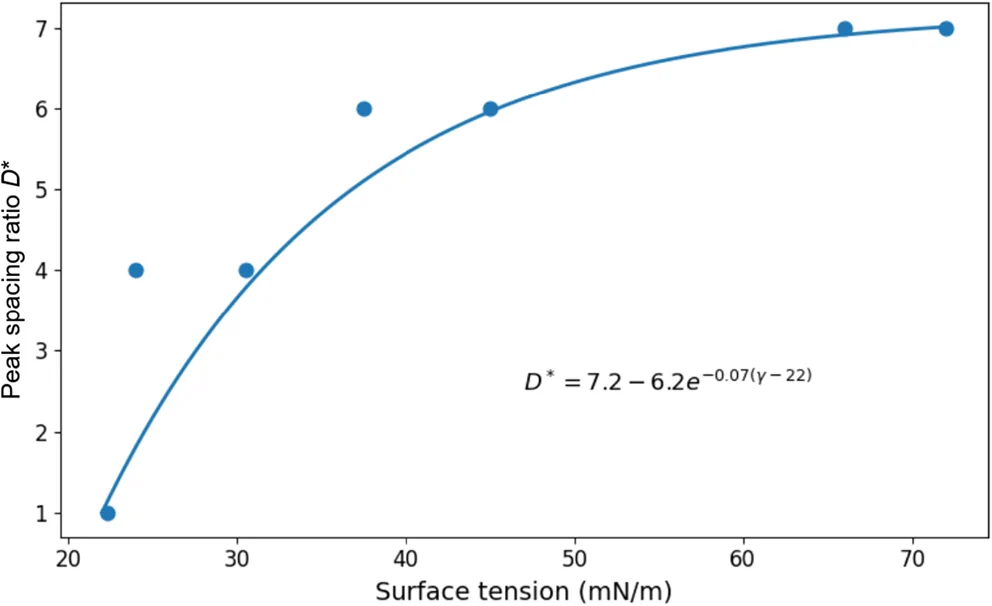
Critical Spacing Ratio Corresponding to Maximum Apparent Contact Angle as a Function of Surface Tension.

The fitted curve provides a continuous design guideline correlating surface tension to the optimal particle spacing (*D*_peak_^*^) required for maximum liquid repellency (eq 6).

6 $$D_{peak}^{*}=7.2-6.2e^{-0.07\left(\gamma -22\right)}$$

While the experimental data identify discrete peak spacing ratios for specific liquids, the curve fit converts these discrete observations into a predictive design line. This relationship shows that as liquid surface tension decreases, the spacing ratio required to maximize the apparent contact angle is reduced. Physically, this trend reflects the significance of geometric design and capillary stabilization for high wetting liquids

To quantitatively define the requisite *P*_*c*_ thresholds for the maximum apparent contact angle and the Cassie-to-Wenzel transition point, the well-known Cassie–Baxter robustness parameter A* is used and calculated according to eq 7.^32^ This allows us to systematically evaluate the thermodynamic stability of the Cassie–Baxter state as a function of the interparticle spacing ratio.

7 $$A^{*}=\frac{l_{cap}}{R\cdot \left(D^{*}-1\right)}\cdot \frac{1-\cos\theta_{intrinsic}}{D^{*}-1+2\;\sin\theta_{intrinsic}}$$

Where *R* denotes the radius of the silica particles (∼500 nm), consistent with the earlier geometric definition. *l*_cap_ is the capillary length representing the characteristic length scale at which surface tension balances gravitational forces. *l*_cap_ is defined by eq 8.

8 $$l_{cap}=\sqrt{\frac{\gamma_{LV}}{\rho \cdot g}}$$

where the γ_LV_ is the surface tension of the tested fluids, ρ is the density of the liquid, and *g* is the gravitational constant of 9.81 m/s^2^. The *l*_cap_ for each liquid used in this work is calculated and listed in Table 1.

**Table 1:** Capillary Length of Test Liquids

Surface tension (mN m^‑1^)	*l*_cap_ (mm)
71.93	2.71
66.42	2.60
30.21	1.76
23.94	1.56
22.37	1.51

Plot of calculated Cassie–Baxter robustness parameter (A*) vs spacing ratio is shown in Figure 7, which demonstrates that for all evaluated ethanol–water mixtures in this work, the Cassie–Baxter robustness parameter (A*) strictly undergoes a monotonic decrease as the spacing ratio (D*) expands. This confirms that increased interparticle spacing progressively compromises the geometric resistance against liquid intrusion; wider gaps reduce the structural confinement of the liquid–vapor interface, precipitating a drop in the critical breakthrough pressure. While robustness decays monotonically, the apparent contact angle exhibits a nonmonotonic profile characterized by a distinct maximum (Figure 5). This delineates a fundamental physical distinction: robustness dictates mechanical structural resistance, whereas the apparent contact angle represents the thermodynamic equilibrium governed by the competition between spatial geometry and wetting stability. On conventional architectures, such as flat-topped micropillars (Figure 8A), the liquid–solid–air triple line is rigidly pinned at the sharp geometrical edges. Consequently, expanding the interpillar spacing strictly reduces the solid fraction (φ_*S*_), driving a continuous increase in the apparent contact angle per the classical Cassie–Baxter model. In contrast, our arrays utilize spherical particles that possess continuously varying topographical angles and strictly lack sharp pinning edges (Figure 8B). This geometry permits the triple line to be easily unpinned and moved along the spherical surface, rendering the effective solid fraction and localized geometric angles highly complex functions of both D* and the intrinsic contact angle. Thus, the initial rise in the apparent contact angle in Figure 5 is driven by the geometrically reduced solid fraction, occurring despite the concurrent, continuous decline in structural robustness (A*). The global maximum of the contact angle curve marks the precise threshold where A* becomes insufficient to support the meniscus. Beyond this critical point, the triple line moves downward along the spheres, resulting in localized interfacial sagging and/or penetration, an increased solid–liquid contact fraction, and a corresponding decay in the apparent contact angle. It should be emphasized that these robustness trends were derived exclusively from polar ethanol–water mixtures and have not yet been experimentally validated for nonpolar liquids such as oils.

**Figure 7:**
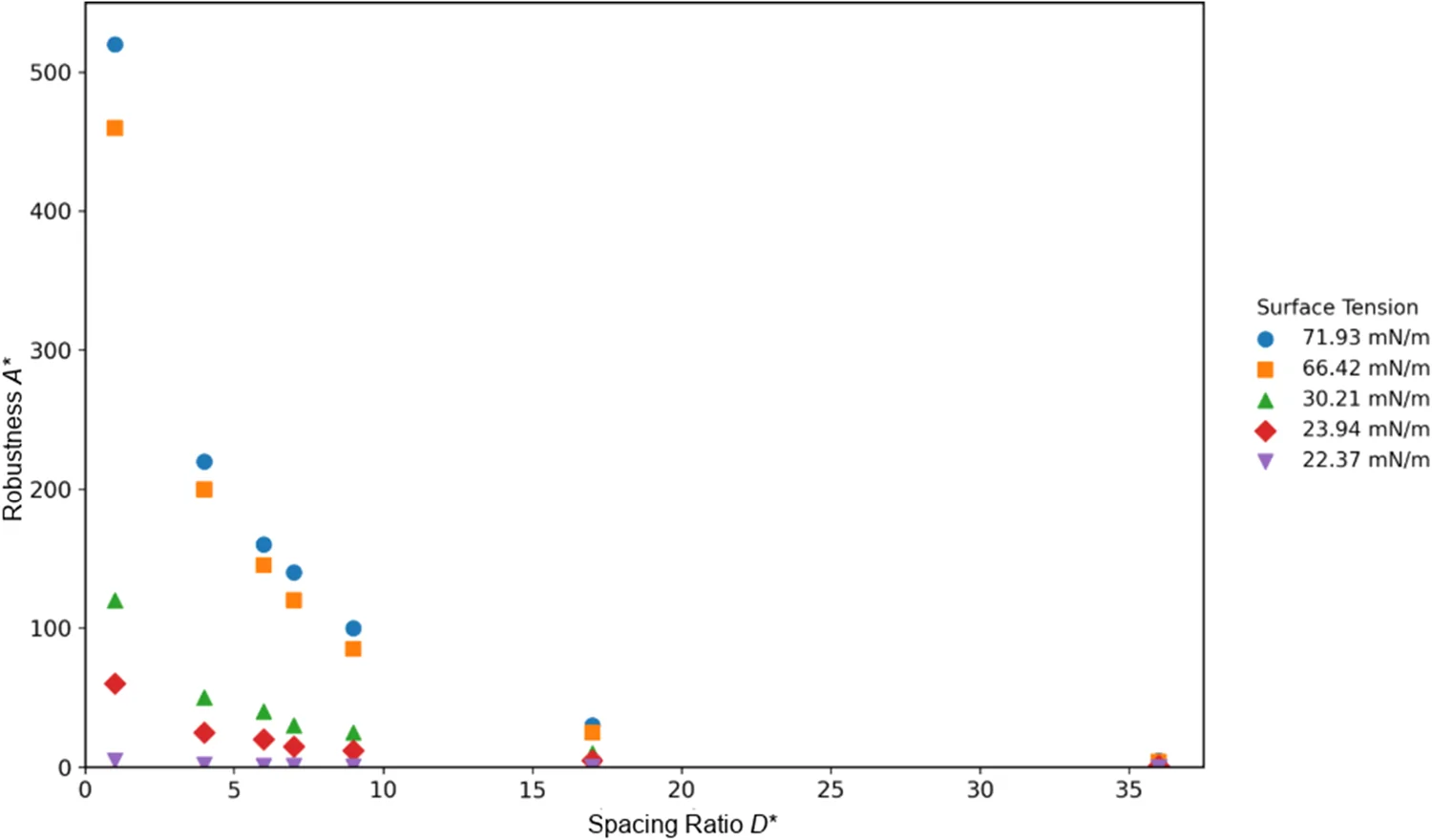
Dimensionless Robustness Parameter A* as a Function of Spacing Ratio for Polar Ethanol–Water Mixtures with Different Surface Tensions.

**Figure 8:**
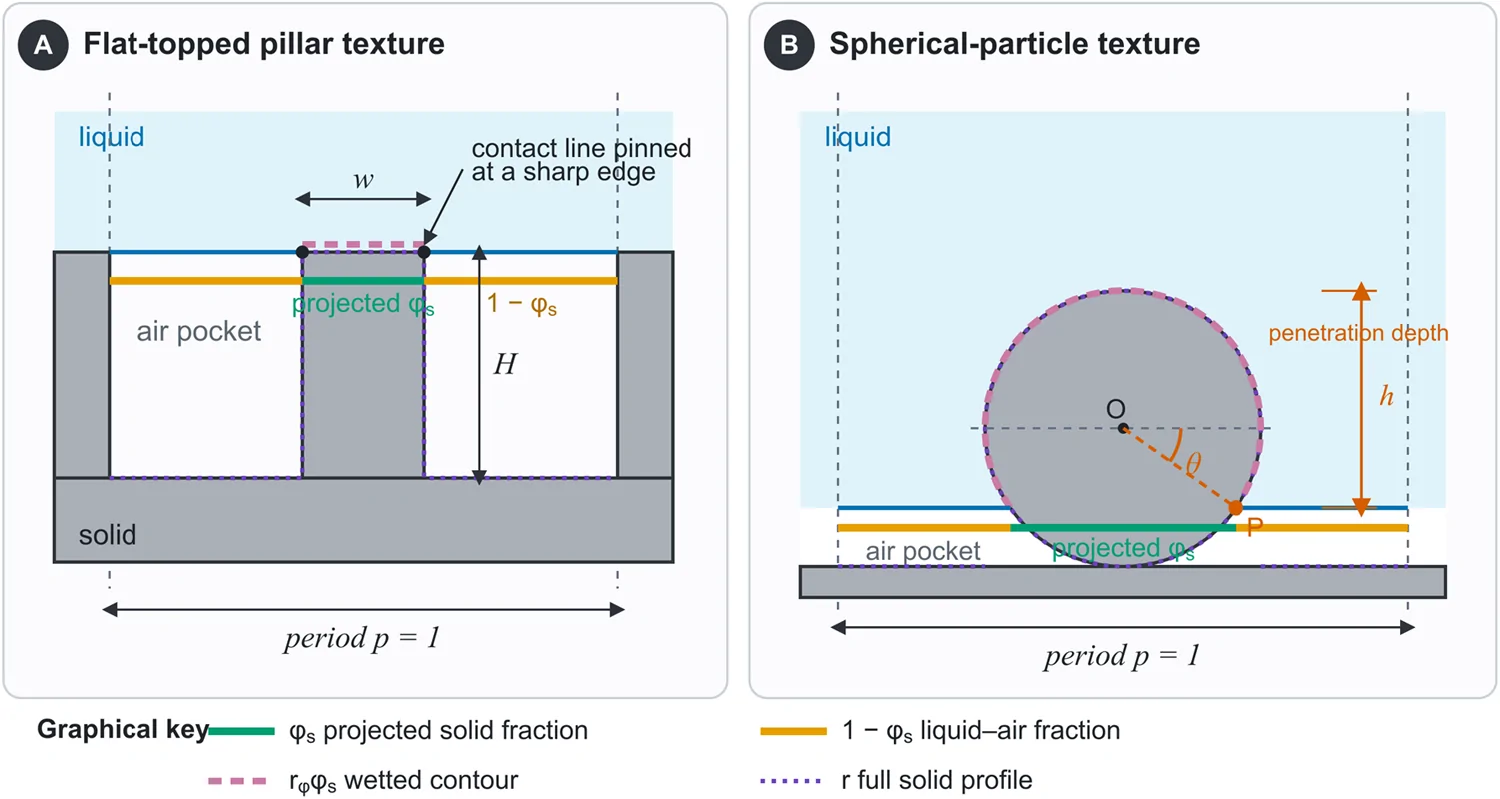
Original schematic comparison of the geometric quantities used in the Wenzel and Cassie–Baxter descriptions for (A) a flat-topped pillar array and (B) a spherical re-entrant texture. The projected unit-cell period *p* is normalized to unity; φ_*s*_ is the projected solid fraction, 1 – φ_*s*_ is the liquid–air fraction, *r*_φ_φ_*s*_ is the actual wetted-solid area normalized by projected area, and *r* is the true-to-projected solid-area ratio. In panel (A), *w* and *H* denote the pillar width and height, respectively. In panel (B), *P* denotes the three-phase contact line, θ is the geometric angle between the horizontal radius from the particle center *O* and the radius *OP*, and *h* is the vertical penetration depth from the particle apex to the liquid–air interface at P.

Additional advancing and receding contact-angle measurements are provided in the Supporting Information. At large spacing, the receding angle decreases strongly relative to the advancing angle, indicating increased contact-line pinning; a qualitative 5 μL tilt test also showed no roll-off up to 90°.

To further elucidate this structural transition, Figure 9 maps the wetting behavior of the 30.21 mN m^–1^ liquid, superimposing schematic insets onto the data points (as shown in Figure S2) to visually define the localized thermodynamic wetting states.

**Figure 9:**
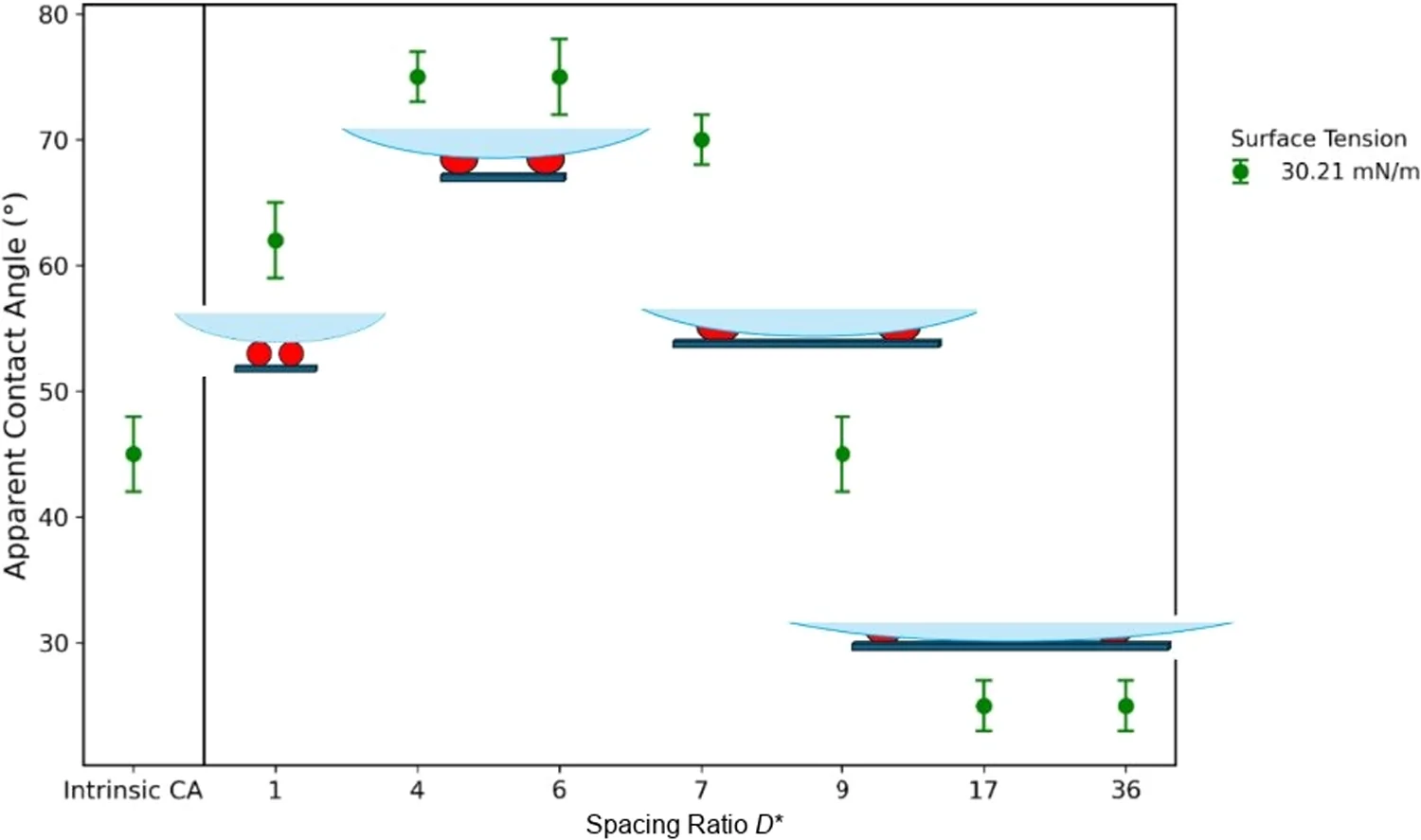
Schematic Illustration of Wetting Transition on Spherical Structures.

To systematically quantify the robustness thresholds (A*) governing both the optimization of the Cassie–Baxter state for the tested polar ethanol–water mixtures (highest apparent contact angle) and the transition to the Wenzel regime, the apparent contact angle and the robustness parameter are coplotted against the spacing ratio (D*) in Figure 10. By correlating the critical D* at the global maximum of the apparent contact angle for each liquid, we extracted the corresponding A* threshold. Remarkably, this optimal structural robustness converges to approximately 100 across all high surface tension liquids. Additionally, by identifying the critical D* at which the apparent contact angle falls below the intrinsic baseline - signifying full Wenzel state - we determined that the critical A* threshold for complete structural failure is approximately 10 for most of the tested liquids.

**Figure 10:**
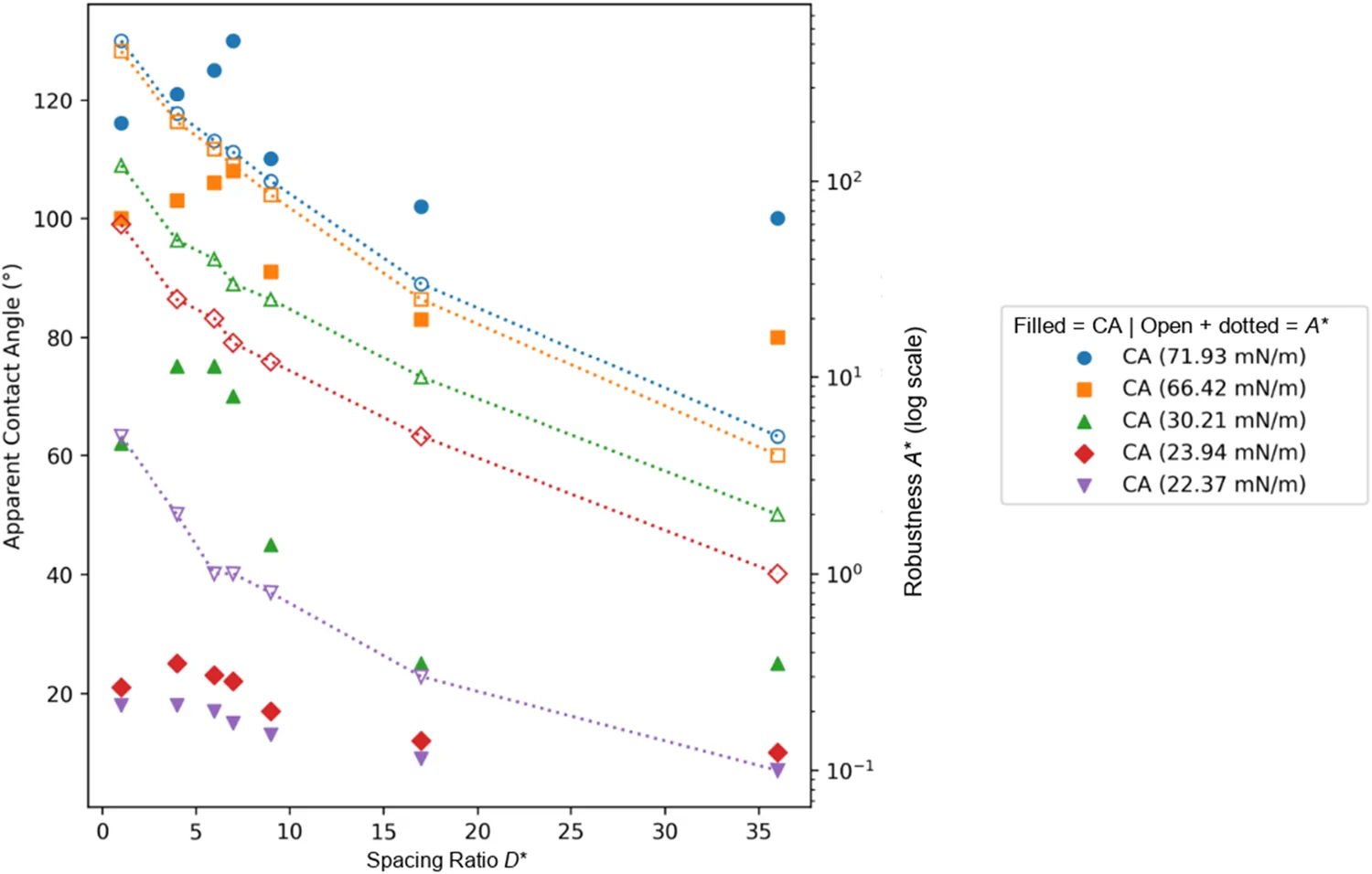
Apparent Contact Angle (CA) and Cassie–Baxter Robustness Parameter (A*) as Functions of Spacing Ratio for Polar Ethanol–Water Mixtures.

These A* values are treated as empirical thresholds for the present 1 μm particle system and tested polar liquids, rather than as universal constants.

## Conclusion

In this work, we established a geometry-based framework for the rational design of PFAS-free liquid-repellent surfaces by identifying interparticle spacing as an independent parameter governing wetting-state stability. Using 1 μm silica particles immobilized in an epoxy matrix together with a sacrificial polystyrene spacer strategy, interparticle spacing was systematically varied while particle size, surface chemistry, and structural height were held constant, enabling the isolated investigation of spacing effects. Wetting characterization with ethanol–water mixtures spanning surface tensions from 71 to 23 mN m^–1^, combined with theoretical analysis, revealed that liquid repellency is governed by a fundamental competition between reducing the solid–liquid contact fraction and maintaining a robust metastable Cassie–Baxter state. This geometric trade-off produces a nonmonotonic dependence of the apparent contact angle on spacing ratio and causes the optimal spacing ratio (D*) to shift toward smaller values as liquid surface tension decreases, demonstrating that lower-surface-tension liquids require increasingly stringent geometric confinement to sustain the suspended wetting state. More importantly, our theoretical framework shows that the robustness parameter (A*) provides a quantitative criterion for predicting both Cassie-state optimization and the onset of Wenzel wetting: for the polar liquids investigated, maximizing the apparent contact angle requires A* > 100, whereas preserving a stable Cassie–Baxter state requires A* ≥ 10. Furthermore, for the spherical re-entrant architecture considered here, an intrinsic contact angle of approximately 40° represents the critical geometric boundary below which a robust Cassie–Baxter state can no longer be sustained. Together, these findings establish predictive, geometry-based design rules that directly link surface morphology to wetting-state stability, providing a fundamental framework for engineering high-performance PFAS-free liquid-repellent coatings. Although this study intentionally isolates the effect of spacing using 1 μm particles and polar ethanol–water mixtures, the proposed framework is expected to be broadly applicable to other particle sizes, liquid systems, and textured surface architectures, offering general guidance for the development of environmentally sustainable liquid-repellent materials.

## Supplementary Material



## Data Availability

The data presented in this study is available on request from the corresponding author.

## Notes

Additional schematics; thermal and rheological characterization; SEM and EDX analyses; liquid-composition data; representative sessile-drop images; and advancing and receding contact-angle measurements (PDF)
