# Supplementary material for: Topography-Driven Liquid Repellency in PFAS-Free Spherical-Particle Coating
Source: Langmuir. 2026 Oct 14;42(42):31302–12. doi: 10.1021/acs.langmuir.6c03964 (PMC13718597; doi:10.1021/acs.langmuir.6c03964)
Supplement: Supplementary file 1 [file la6c03964_si_001.pdf]

SUPPORTING INFORMATION

Topography-Driven Liquid Repellency in PFAS-Free Spherical-Particle Coating

Talya A. Scheff [1], *Jinde Zhang*[1*], *Hanna Dodiuk* [2], *Samuel Kenig* [2], *Margaret A Auerbach* [3], *Timothy J Lawton* [3], *and Joey Mead* [1]

1. University of Massachusetts, Lowell, MA 01854, USA
2. Shenkar College of Engineering and Art, Ramat Gan 5252626, Israel
3. United States Army Combat Capabilities Development Command Soldier Center (DEVCOM SC), Natick, MA 01760, USA

Contents

• Figures S1-S2. Re-entrant geometries and the spacing-driven Cassie-Wenzel transition
• Figures S3-S5. Epoxy curing, gelation, and particle-exposure optimization
• Figure S6. EDX analysis of solvent residues after spacer removal
• Figure S7. Effects of PS:silica ratio and removal solvent on normalized spacing
• Figures S8-S9. EDX carbon and silicon maps before and after PS removal
• Table S1 and Figure S10. Test-liquid compositions and representative sessile-drop images
• Figures S11-S13. Static, advancing, and receding contact-angle measurements
• Qualitative roll-off observation

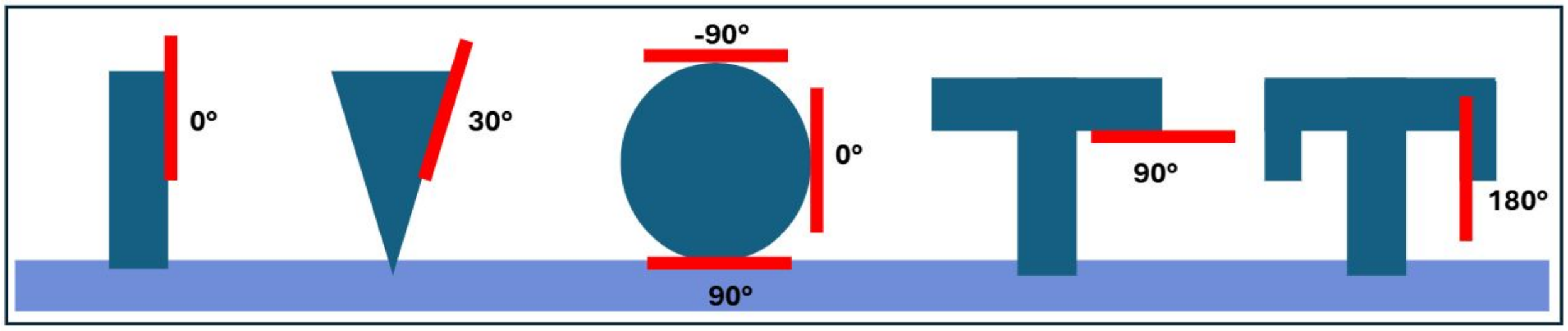

Figure S1. Illustration of Different Re-entrant Structures.

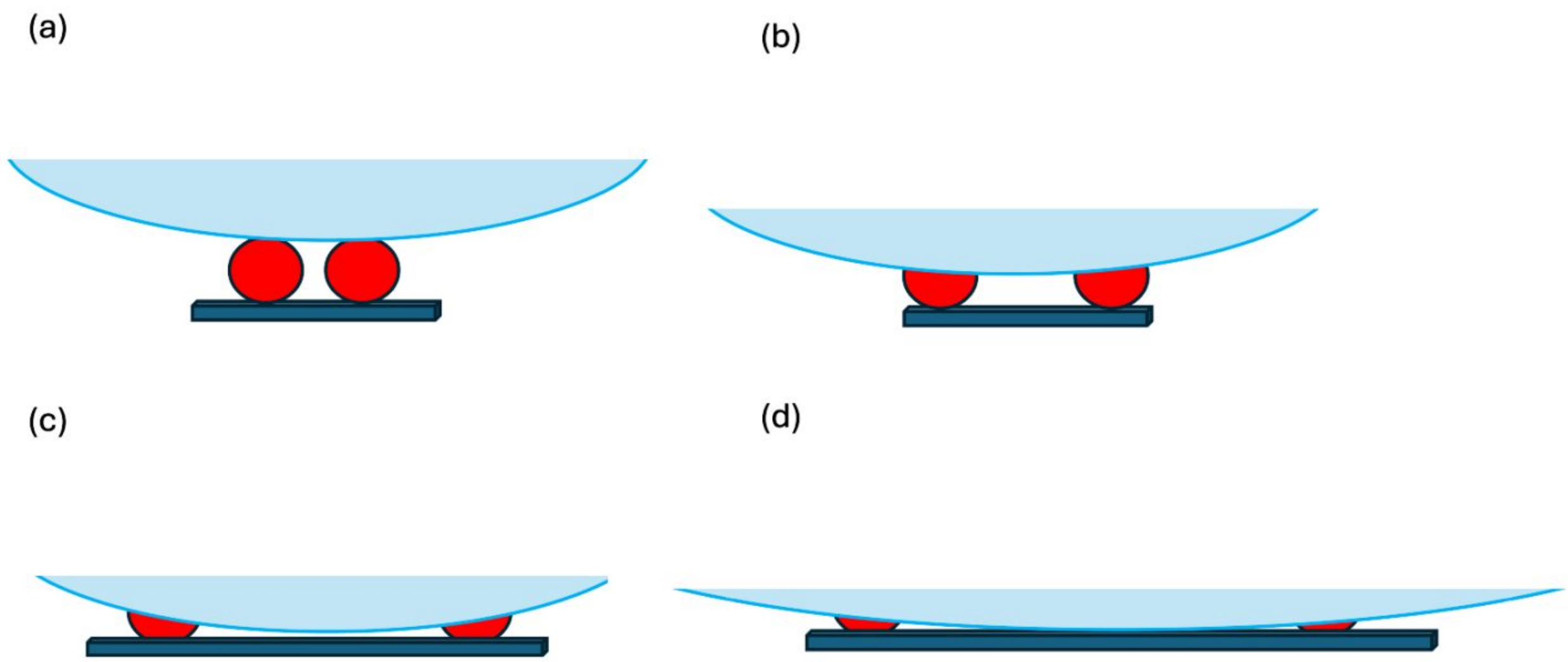

Figure S2. Schematic illustration of the Cassie–Wenzel transition with increasing interparticle spacing: (a) suspended Cassie state; (b) meniscus sagging at intermediate spacing; (c) partial liquid intrusion at larger spacing; and (d) complete intrusion into the texture (Wenzel state).

Optimization of Epoxy Pre-Cure: Controlling Particle Exposure and Fixation

A critical requirement for the systematic investigation of spacing-dependent wettability is the creation of a mechanically stable particle scaffold in which silica spheres are simultaneously (i) maximally exposed to the liquid interface to impose the smallest possible geometric angle (approaching 0˚, as illustrated in Figure 1 of the Introduction) and (ii) stably immobilized on the

substrate to prevent displacement during solvent removal and wetting measurements. This requirement was addressed by optimizing the epoxy pre-cure time prior to particle deposition. The curing behavior of the epoxy was first characterized using differential scanning calorimetry (DSC, **Figure S3**) and oscillatory rheology (**Figure S4**). The DSC thermogram shows a pronounced exothermic curing peak for the uncured epoxy, confirming that crosslinking occurs during heating and that complete curing is achieved after 110 ˚C for 2 h. Complementary time-sweep rheology measurements reveal a rapid increase in viscoelastic moduli as curing progresses, with the gel point occurring at approximately 4 min. At this transition, the material changes from a viscous liquid to a viscoelastic solid network, indicating a sharp increase in viscosity. This rheological transition is critical for the particle deposition step because epoxy viscosity directly controls the embedding depth of silica particles deposited onto the surface.

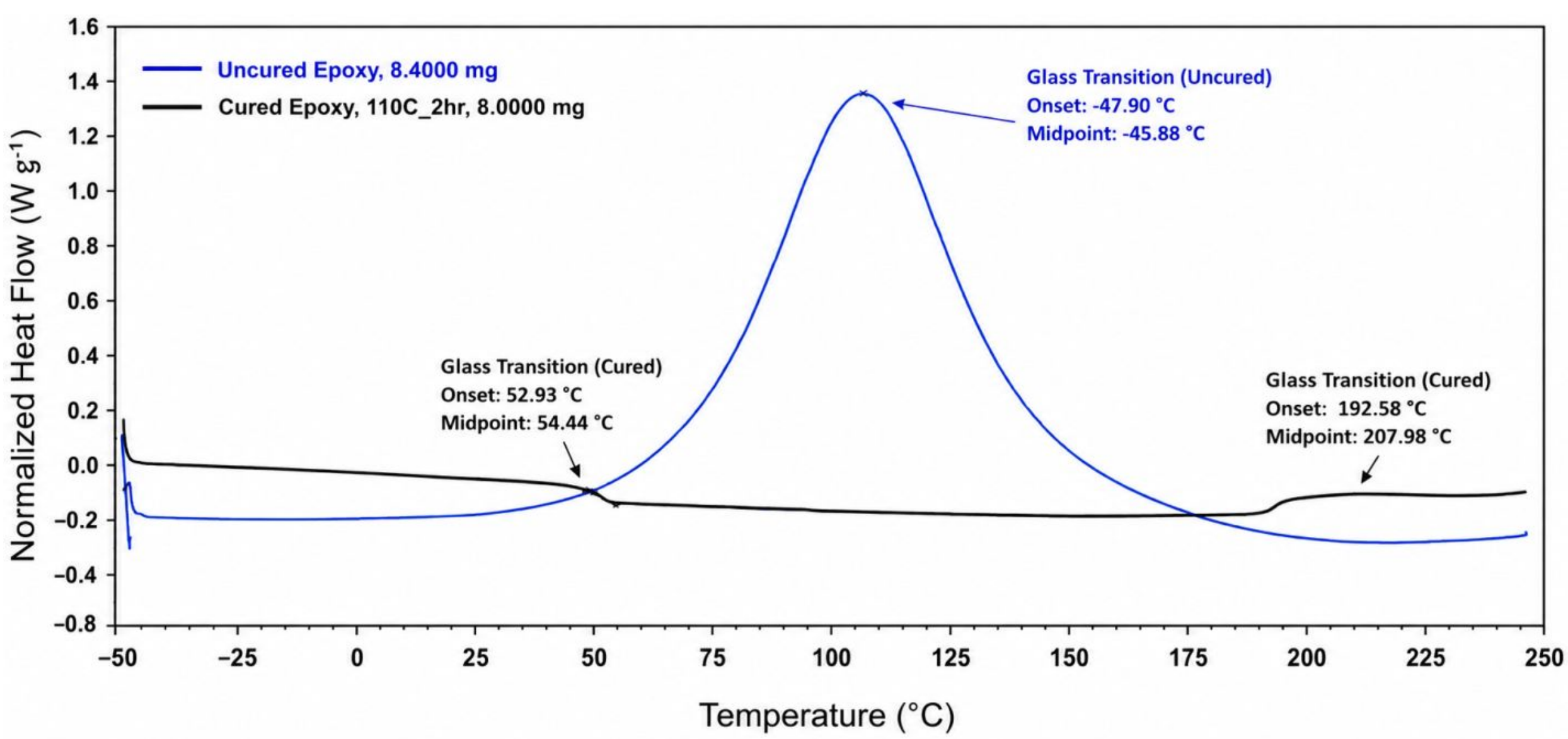

Figure S3. Differential Scanning Calorimetry (DSC) Thermogram of Epoxy Cure Behavior

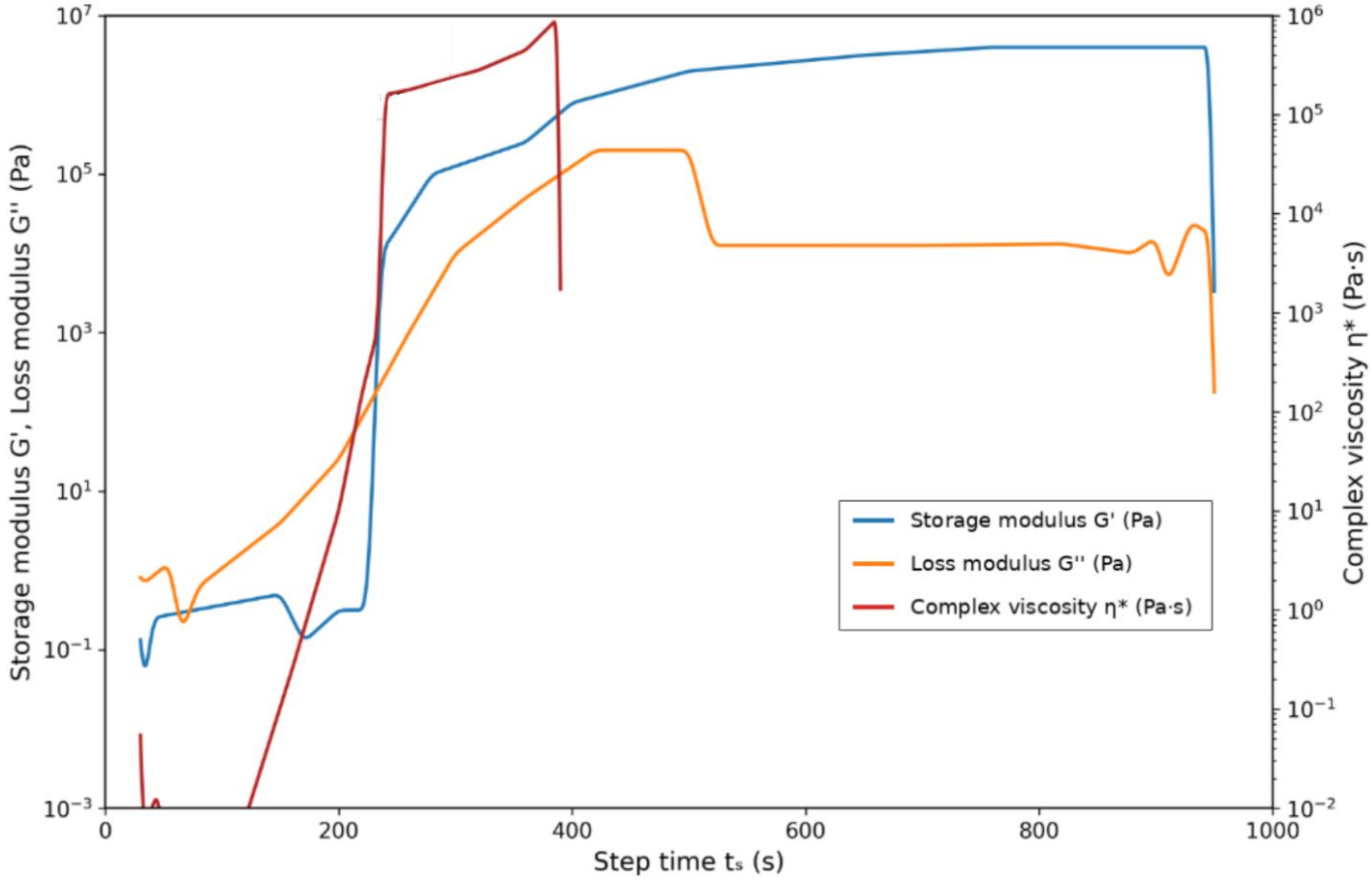

Figure S4. Time-Sweep Rheological Evolution of Epoxy during Curing and Gelation

Therefore, the epoxy layer was pre-cured at 110 ˚C for various times ranging from 4–7 min. prior to dip-coating the 1 µm silica spheres. Because viscosity increases as curing approaches and passes the gel point, the pre-cure time determines the degree to which particles sink into the binder. SEM micrographs of the resulting surfaces (**Figure S5**) show that at 4 min, approximately corresponding to the gel time, the epoxy remains sufficiently fluid to allow significant particle embedding, resulting in an apparent particle diameter smaller than the nominal 1 µm. Increasing the pre-cure time reduces particle penetration as viscosity rises, progressively increasing the exposed height of the spheres. When pre-cure time exceeds 5 minutes (**Figure S5 b, c and d**), most particles exposed

out. Ultimately, a 6-minute pre-curing time was selected for all experiments to ensure consistency and process efficiency.

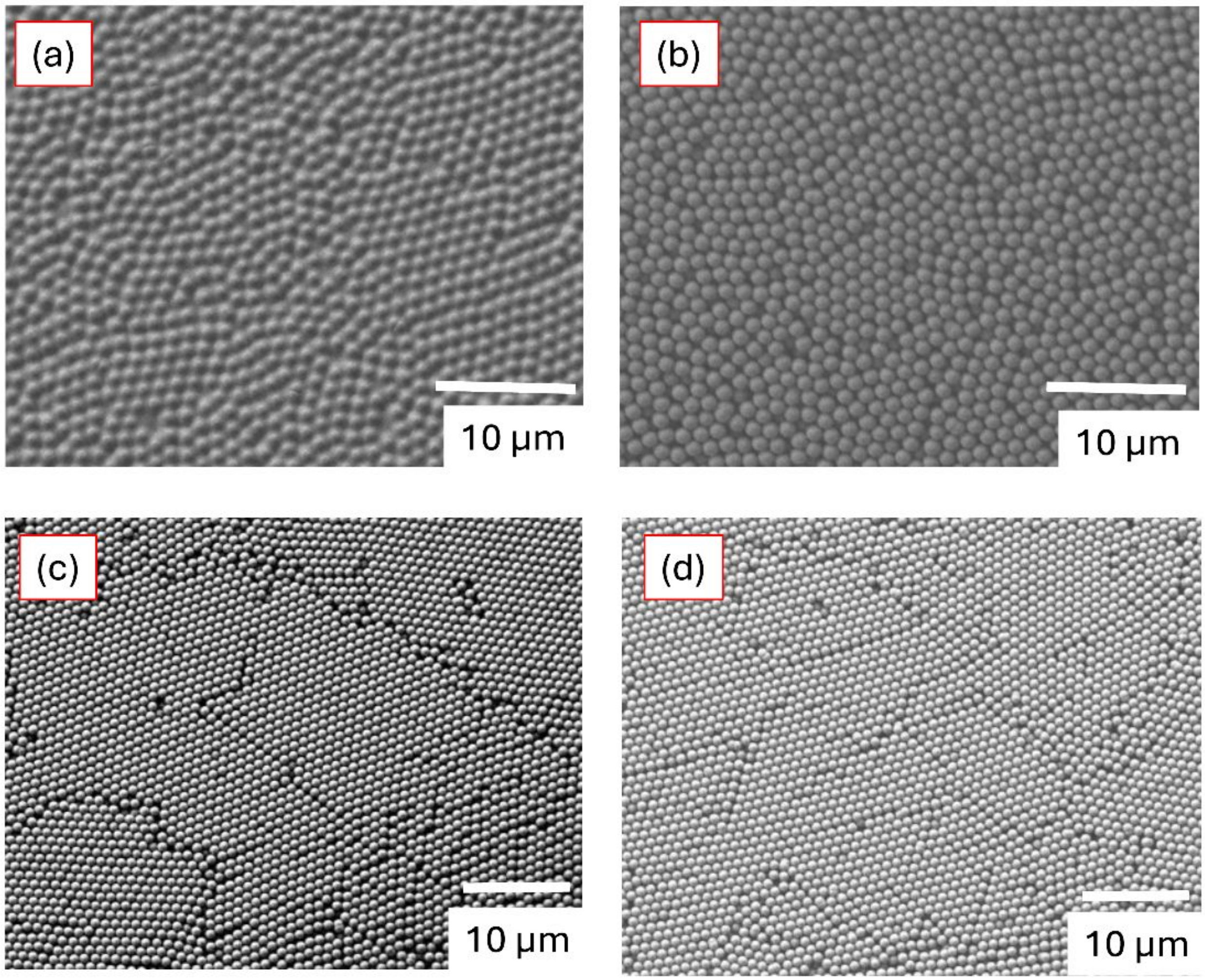

Figure S5. SEM Images Showing Effect of Epoxy Pre-Cure Time on Particle Embedding and Exposure (a) 4 min, (b) 5 min, (c) 6 min, (d) 7 min

EDX Analysis of Solvent Residues

To determine whether the solvent treatment used to remove the sacrificial polystyrene phase also caused the detachment of silica spheres, the solvent collected after immersion was analyzed. The

wash solution was deposited onto a clean glass substrate, allowed to dry, and the remaining material was examined by EDX, as seen in **Figure S6**. The elemental maps show discrete spherical features that appear in yellow, corresponding to the silicon signal. These yellow spherical regions confirm the presence of silica particles that were washed off during the solvent treatment. EDX also revealed that using a mixture of hexane:toluene instead of pure toluene resulted in removal of the PS spacer and reduced loss of loosely bound silica particles, yielding smaller interparticle distances compared to samples treated with toluene alone. This additional parameter provided enhanced control over the final morphology, enabling more precise tuning of *D** within the targeted range.

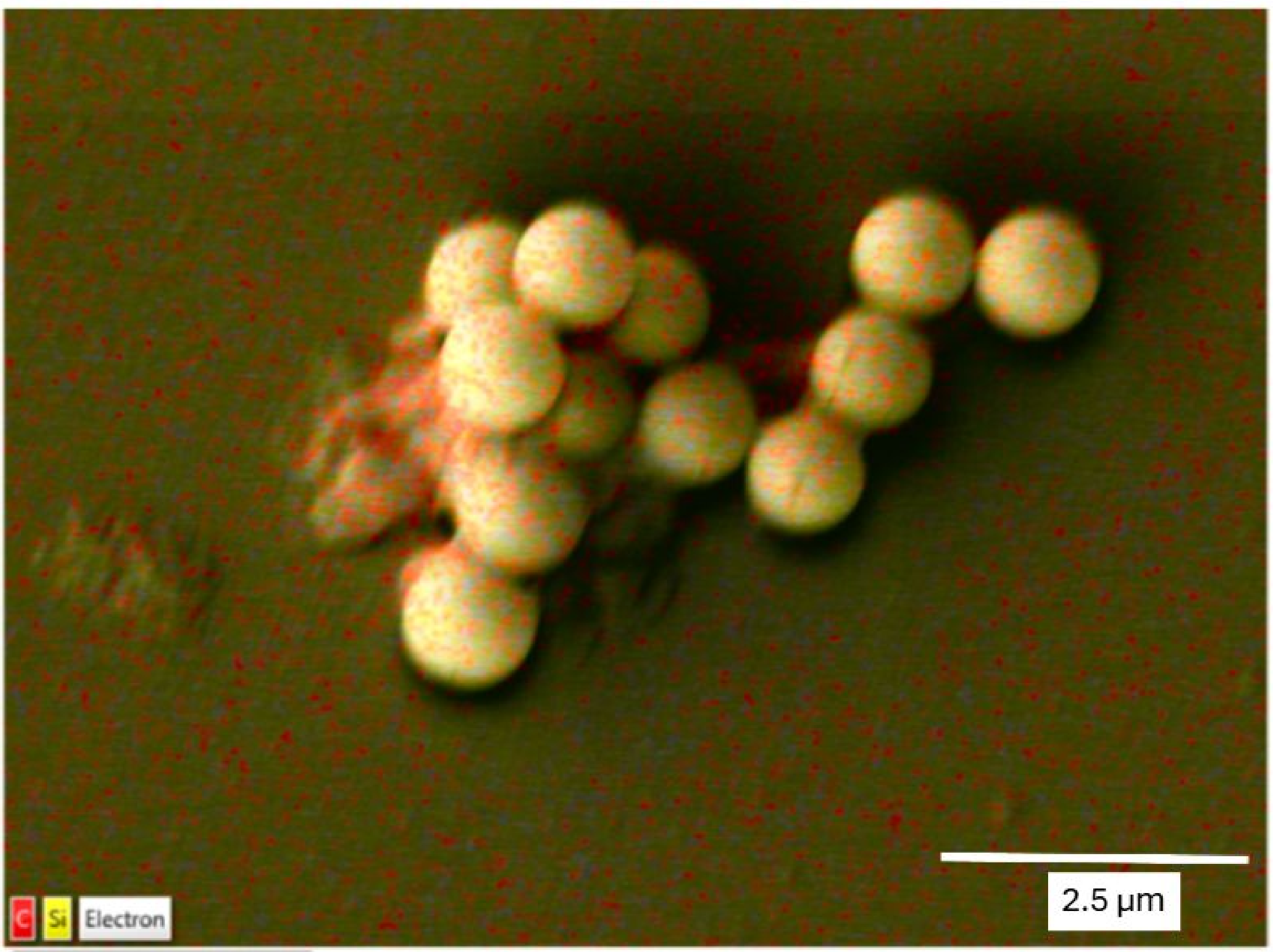

Figure S6. EDX Analysis of Solvent Residue Demonstrating Silica Particle Loss during Spacer Removal

Effect PS:Silica ratio on D*

The effect of the polystyrene (PS) sacrificial spacer content on the interparticle spacing of silica particles was systematically investigated by varying the PS:silica ratio prior to deposition. A baseline condition without a spacer (0:1 PS:silica) resulted in a closely packed morphology, as shown in **Figure S7a**, where silica particles are in near-direct contact with minimal interparticle spacing. Introducing PS at a 1:1 ratio, as shown in **Figure S7b**, followed by removal of the PS after curing using toluene, generated sacrificial spacer gaps between adjacent silica particles, confirming the role of the spacer in defining particle separation.

Further increases in the PS content (2:1, 3:1, 4:1, 5:1, and 10:1 PS:silica ratios), as shown in **Figure S7d, f, g, h and i** respectively, led to a progressive increase in the interparticle distance. As the amount of sacrificial spacer increased, a systematic expansion of spacing between silica particles was observed, demonstrating that the PS content provides a direct and controllable means of tuning the spacing ratio (D^*). This approach enabled the generation of a wide range of surface morphologies, from closely packed to highly separated particle arrangements, thereby establishing a controlled platform for investigating the effect of spacing on wetting behavior.

To achieve finer control over interparticle spacing beyond what could be obtained by adjusting the PS:silica ratio the solvent used for spacer removal was varied as discussed above in **Figure S6.** Specifically, for selected compositions (2:1 and 3:1 PS:silica) as shown in **Figure S7c and e, a**

mixed solvent system of hexane:toluene was used instead of pure toluene, which yielded smaller interparticle distances compared to samples treated with toluene alone. This additional parameter provided enhanced control over the final morphology, enabling more precise tuning of D^* within the targeted range.

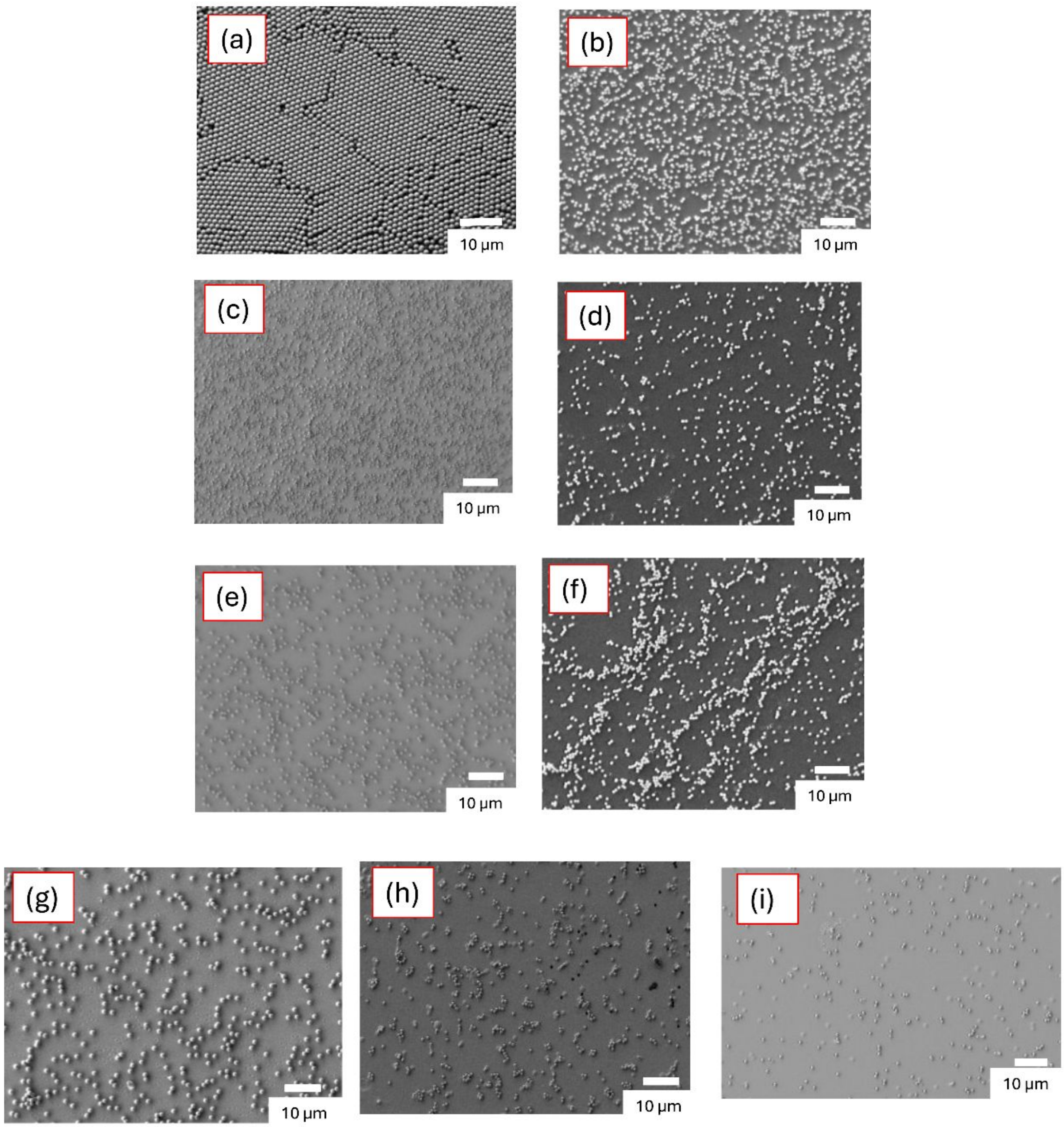

Figure S7. SEM Images Showing Effect of systematically varying PS: silica ratio and spacer removal solvent :Toluene or Hexane/Toluene mixture (a) 0:1, (b) 1:1 using Toluene, (c) 2:1 using Hexane/Toluene mixture. (d) 2:1 using Toluene, (e) 3:1 using Hexane/Toluene mixture, (f) 3:1 using Toluene, (g) 4:1 using Toluene, (h) 5:1 using Toluene and (i) 10:1 using Toluene.

EDX Carbon and Silicon Signal results

Figures S8a–d present the compositional and morphological characterization of the sample prior to toluene spacer removal using energy-dispersive X-ray spectroscopy (EDX) coupled with SEM. The carbon (C) signal (**Figure S8a**) corresponds to the polystyrene (PS) sacrificial spacer, while the silicon (Si) signal **(Figure S8b**) represents the silica particles. The corresponding SEM image (**Figure S8c**) and the overlay of the EDX elemental maps with the SEM micrograph (**Figure S8d**) confirm the co-localization of PS and silica within the composite structure.

Figures S9a–d show the same set of analyses after toluene treatment for spacer removal. A pronounced reduction in the carbon signal is observed, indicating effective removal of the PS spacer, while the silicon signal remains unchanged, confirming the retention and distribution of the silica particles. The overlay images further illustrate the disappearance of carbon-rich regions and the resulting increase in interparticle spacing following spacer removal. The carbon signal shown in **figure S9d** corresponds to the carbon elements present in the epoxy binder layer on the surface.

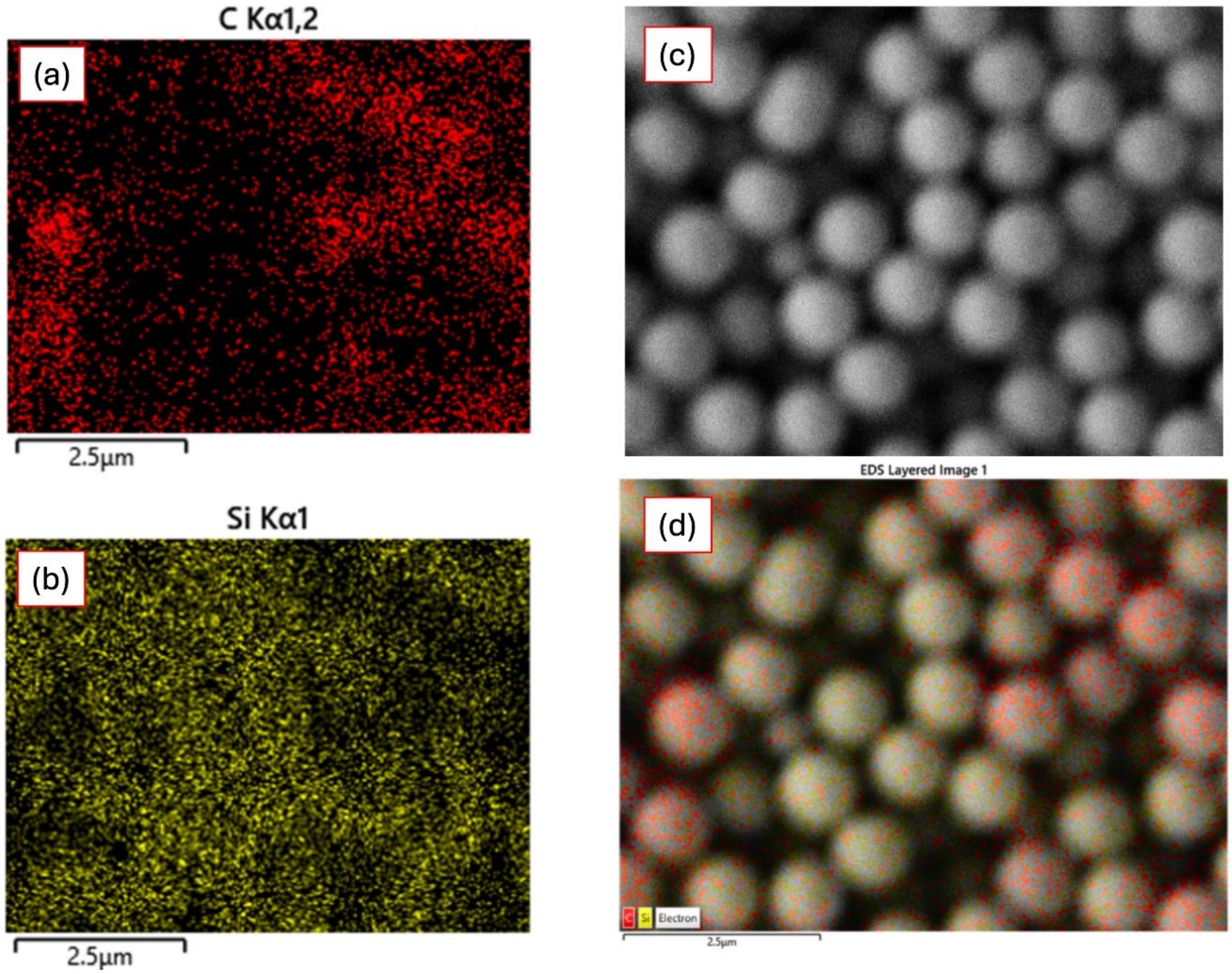

Figure S8. (a) EDX carbon (C) map, (b) silicon (Si) map, (c) SEM image, and (d) EDX overlay of a silica/PS composite before toluene spacer removal

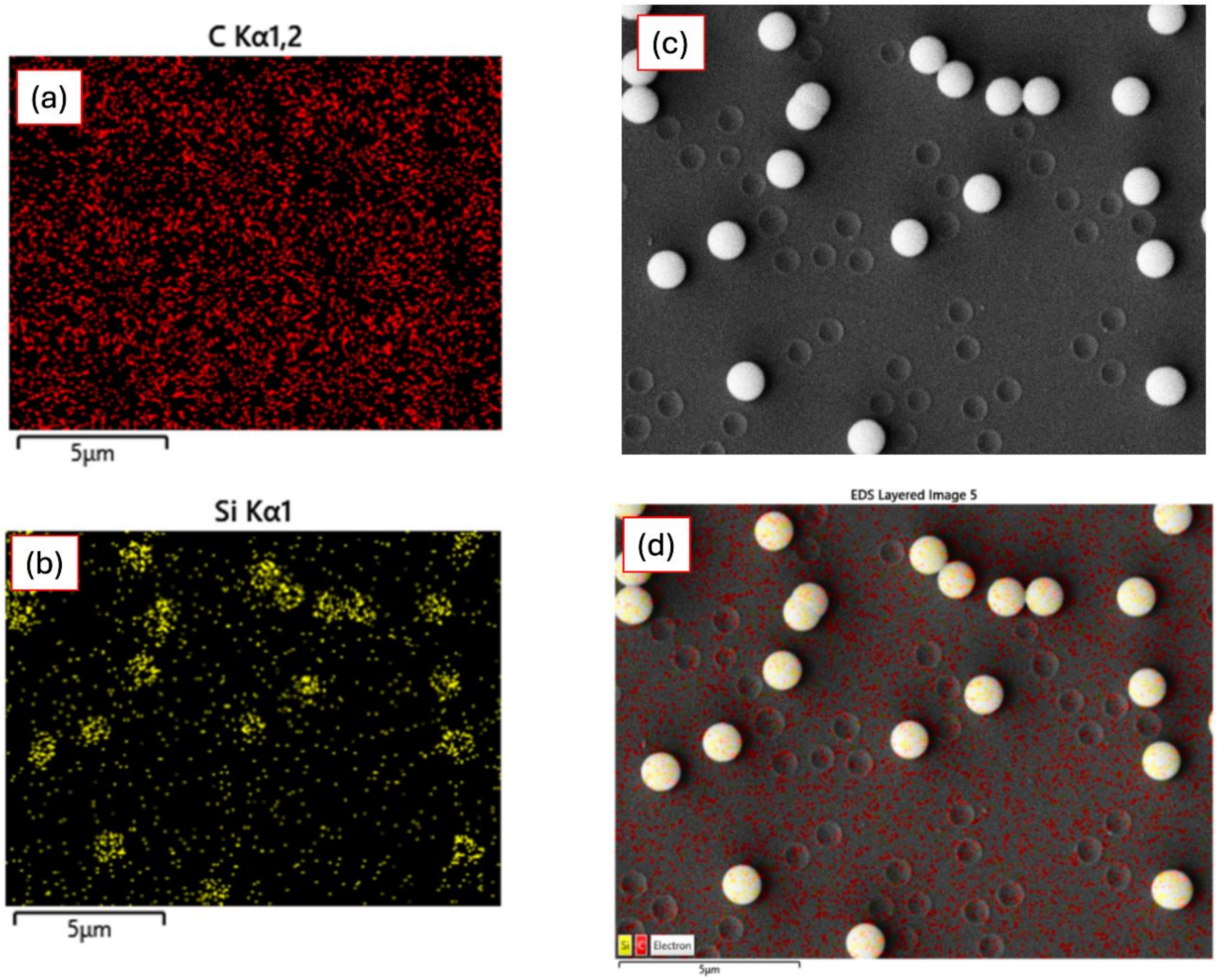

Figure S9. (a) EDX carbon (C) map, (b) silicon (Si) map, (c) SEM image, and (d) EDX overlay of a silica/PS composite after toluene spacer removal

Additional Wettability Measurements

The water/ethanol mixtures were prepared by weight using literature compositions corresponding to the target surface tensions used in the main manuscript. The target values were checked experimentally by pendant-drop analysis at room temperature before contact-angle measurements; the figures in the manuscript are labeled using the literature target surface tensions.

Table S1. Water/ethanol compositions and literature target surface tensions used for wettability testing.

Water (wt.%)	Ethanol (wt.%)	Target surface tension (mN m^{-1})
100.00	0.00	71.93
99.24	0.76	66.42
61.45	38.55	30.21
10.67	89.33	23.94
1.20	98.80	22.37

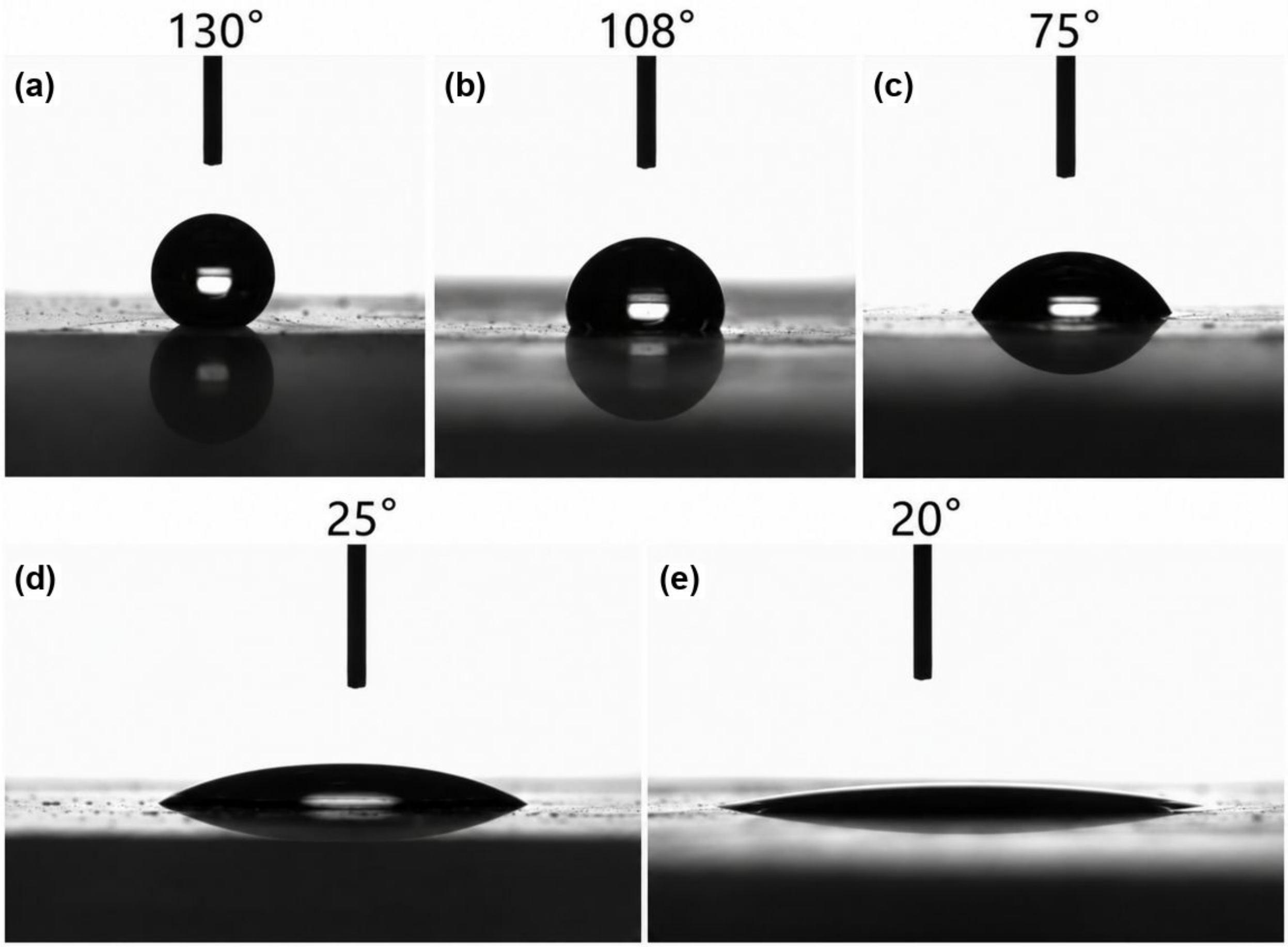

Figure S10. Representative sessile-drop images at the spacing ratio corresponding to the peak static contact angle for each tested liquid: (a) $D^* = 7$, 100.00/0.00 wt% water/ethanol ($\gamma = 71.93$ mN m^{-1}); (b) $D^* = 7$, 99.24/0.76 wt% water/ethanol ($\gamma = 66.42$ mN m^{-1}); (c) $D^* = 4$, 61.45/38.55 wt% water/ethanol ($\gamma = 30.21$ mN m^{-1}); (d) $D^* = 4$, 10.67/89.33 wt% water/ethanol ($\gamma = 23.94$ mN m^{-1}); and (e) $D^* = 1$, 1.20/98.80 wt% water/ethanol ($\gamma = 22.37$ mN m^{-1}). The measured apparent contact angle is shown above each image.

Advancing and Receding Contact Angles

Advancing and receding contact angles were measured to assess contact-line mobility and pinning. The advancing angles remain close to the static values over much of the spacing range, whereas the receding angles fall sharply as $D*$ becomes large. Consequently, contact-angle hysteresis increases substantially after the optimum-spacing region, consistent with increasing liquid penetration and pinning. These measurements support the proposed wetting transition but are not treated as direct proof of trapped air at the interface.

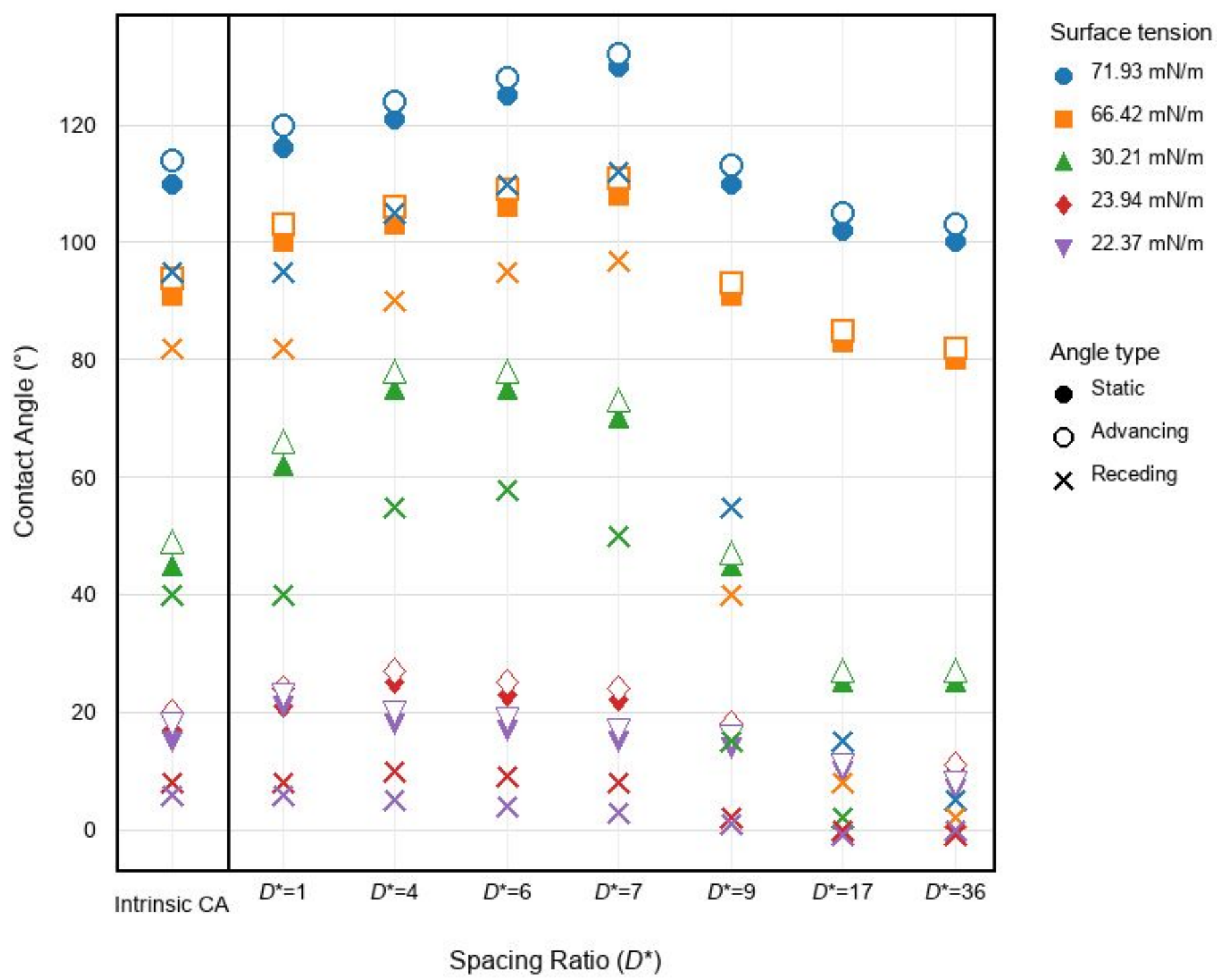

Figure S11. Static (filled markers), advancing (open markers), and receding (× markers) contact angles as a function of normalized spacing ratio $D*$ for the tested water/ethanol mixtures. The vertical line separates the smooth intrinsic-contact-angle reference from the structured samples.

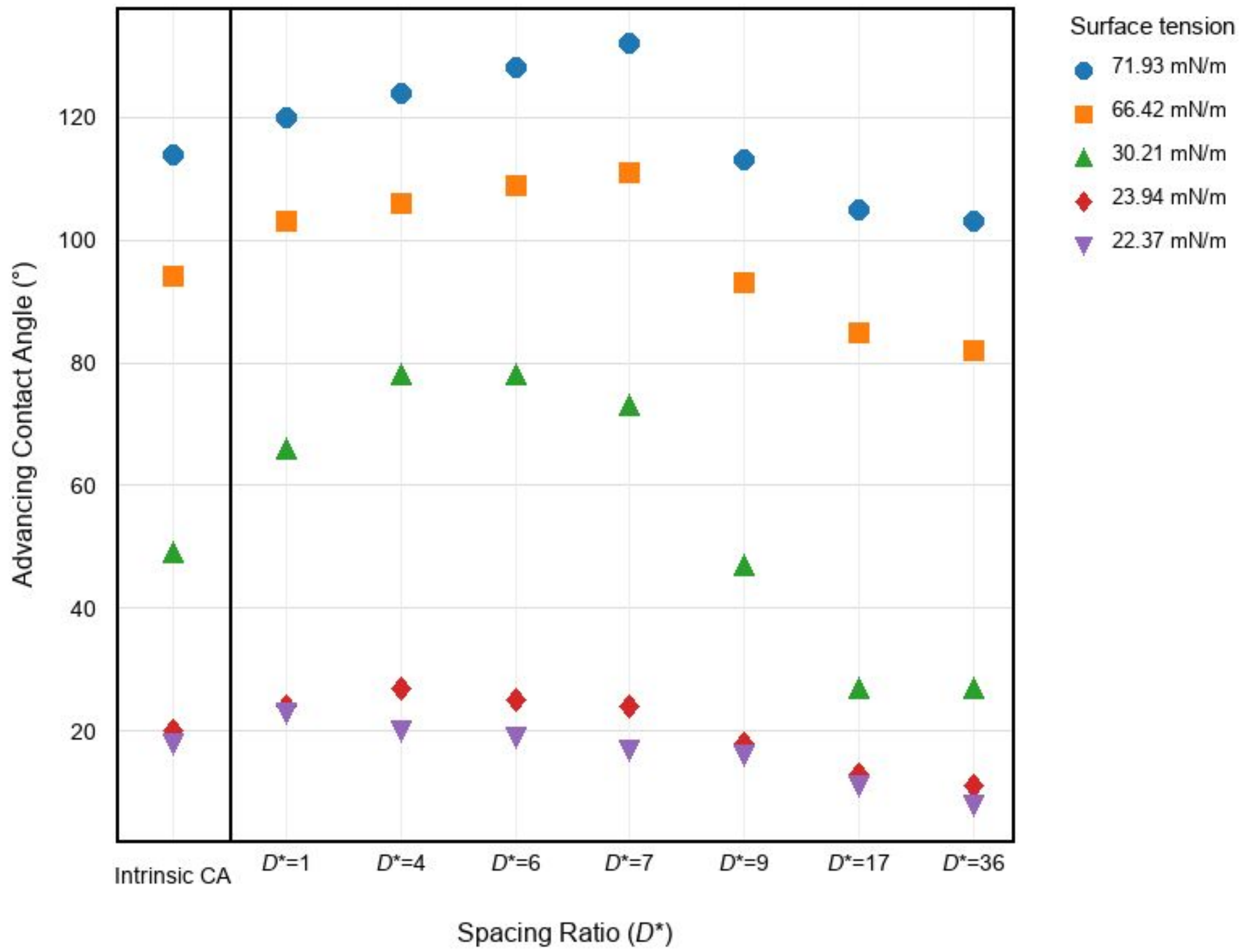

Figure S12. Advancing contact angle as a function of normalized spacing ratio D^* for the tested water/ethanol mixtures. The vertical line separates the smooth intrinsic-contact-angle reference from the structured samples.

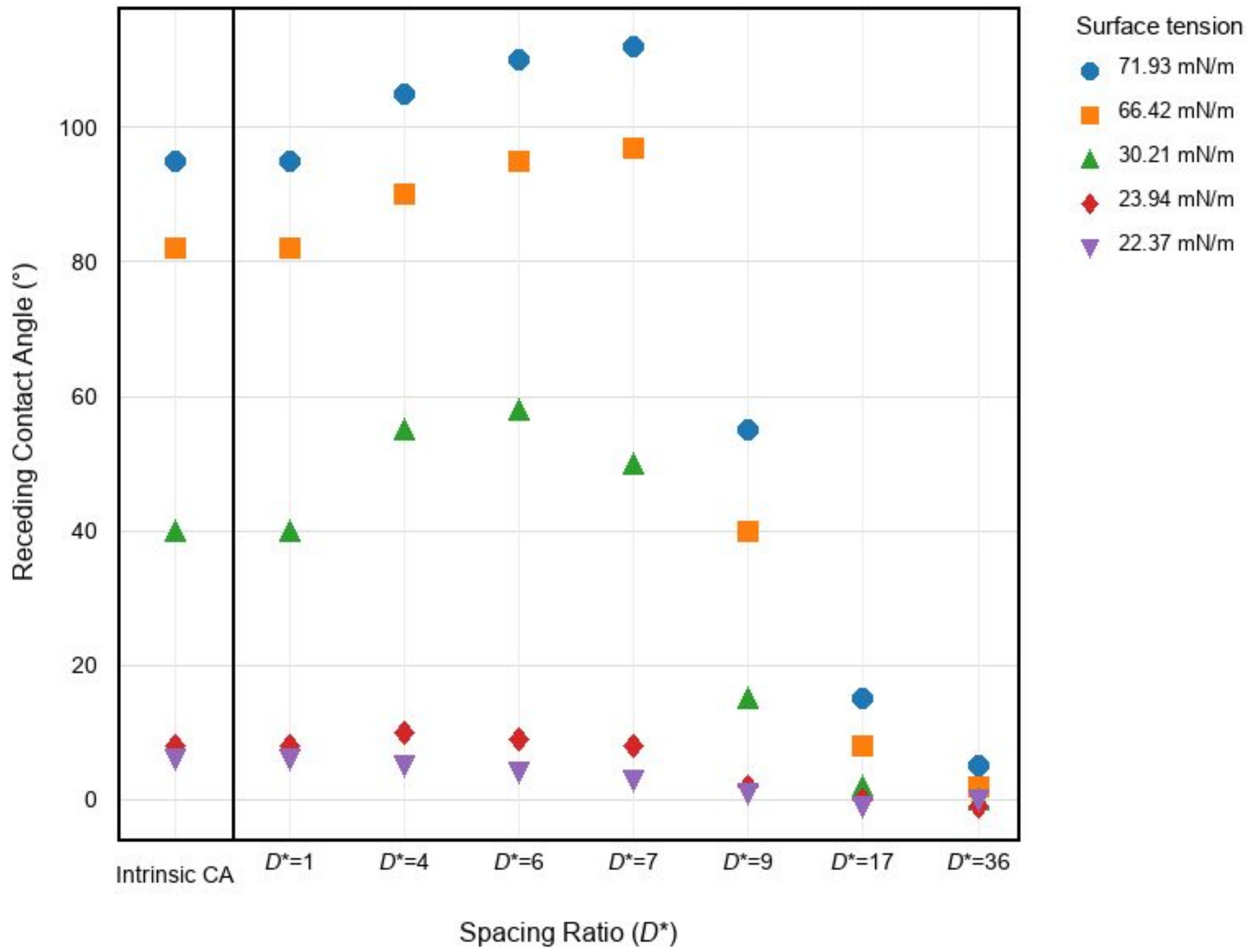

Figure S13. Receding contact angle as a function of normalized spacing ratio *D** for the tested water/ethanol mixtures. The vertical line separates the smooth intrinsic-contact-angle reference from the structured samples.

Qualitative roll-off observation. A 5 μL droplet on a representative condition did not roll off when the sample was tilted to 90°. The absence of roll-off is consistent with the very low receding angle and strong pinning observed in the dynamic contact-angle measurements.
